# Supplementary material for: A Modified Methyl Transferase Cofactor to Selectively Silence Gene Expression in Escherichia coli
Source: Chembiochem. 2026 Mar 16;27(6):e202500737. doi: 10.1002/cbic.202500737 (PMC12990290; doi:10.1002/cbic.202500737)
Supplement: Supplementary file 1 — Supplementary Material [file CBIC-27-e202500737-s001.pdf]

A Modified Methyl Transferase Cofactor to Selectively Silence Gene Expression in *E. coli*

Oliver J. Irving*, Samuel Stone, Robert K. Neely, Tim Albrecht*

* Corresponding authors o.irving.1@bham.ac.uk, t.albrecht@bham.ac.uk

School of Chemistry, University of Birmingham, Edgbaston Campus, Birmingham B15 2TT, United Kingdom

Supporting Information

SI1. Methodology

All bacterial work was carried out under aseptic conditions on a 70% ethanol sterilized surface, next to a Bunsen burner, with all equipment sterilized using the ethanol solution and flame.

Plasmid modification

Plasmids were modified using restriction, and ligation insertion of a short dsDNA fragment. The insertion sequence is provided below. The modification method for the short dsDNA fragments is reported in Irving *et al*[25].

Short dsDNA plasmid inserts (500 ng/µL, sequence below, was incubated with 2 µL Cutsmart buffer 10X, 1 µL of the modification donor at 100 µM (CH_3 (SAM), MTC5 (SAH-MTC5), or AdoHcy azide (SAH-AdoHcy azide, AHA), illustrated in the main paper, and used independently), 0.5 µL m.TaqI (NEB) and 15.5 µL of nuclease free water (Merck), adding the enzyme last. This was incubated at 40°C for 1.5 hours. To this, 0.5 µL of proteinase K (800 units/mL, NEB), was added and incubated at 40°C for 1 hour. This was allowed to cool to room temperature for 20 minutes post heating. The solution was then purified using a PCR clean-up kit (GenElute™, Sigma-Aldrich), and stored at 4°C until required.

5’ - TAG TCG AAT CGA GTA CCG - 3’

5’ - TTT CGG TAC TCG ATT CGA – 3’

To insert this sequence into the plasmid (S10-pMA-RQ, pET-28c(+), and pCSDEST2-EGFP) at the gene promoter site, a 50 µL digestion was set up using the parameters described in table S1, and incubated at 37°C for 1 hour, before incubation on ice.

Table S1. EarI restriction reagents.

Reagent	Concentration
DNA	1 µg
10X rCutSmart Buffer	5 µL (1X)
EarI	1.0 µL (20 units)
Nuclease-free Water	to 50 µL

To reassemble the plasmids to contain the modified insert, in a new vial, 50 ng of cut plasmid, 150 ng of modified insert, and 5 µL of Quick Ligation™ Kit (NEB) were added and incubated at room temperature for 15 minutes. Plasmids were stored at -20°C until transformation.

Transformation

Competent *E.coli* cells (DH5α and T7 enhanced BL21, Thermofisher Scientific and NEB respectively) were removed from storage at -80°C and allowed to thaw on ice for 30 minutes. 50 µL of the thawed cells were placed into a new vial and 3 µL of each respective plasmid DNA was added and incubated on ice for 30 minutes. Each vial was then heat shocked at 42°C water bath for 60 seconds. The vials were placed back on ice for 2 minutes before 1 mL of SOC media was added to each vial and incubated on a 37°C shaking incubator for 45 minutes.

Bacterial growth plates

Three bacterial plate conditions were used to determine the presence, and effect, of the plasmid modification on bacterial growth. These conditions were the inclusion and absence of ampicillin and/or kanamycin as a selection factor. Where appropriate these are noted in the results section. LB

agar was created using 40 g/L of LB agar broth (Thermofisher) and distilled water, and autoclaved for 30 minutes. Where appropriate, 100 mg/mL Ampicillin sodium salt (Thermofisher) or 50 mg/mL kanamycin (Merck) was added to the LB agar, once the temperature had reached 50°C. 15 mL of the prepared agar was poured into 7.5 cm petri dishes. The plates were allowed to cool to room temperature for 45 minutes until the agar had completely solidified. 30 µL of each of the transformed bacteria was pipetted onto the agar plate and swept across the surface using a sterilized glass spreader. The plates were incubated at 37°C for up to 48 hours. Growth was determined by the number of colonies.

Bacterial colony growth, examples

The growth plates examples, as shown in figure S1, illustrate the colony formation observed after transformation. The number of colonies were counted using the circular finding tool in Fiji-imageJ and checked manually to ensure reliability. The labelling on the plates are: "Con" for no plasmid, "S10" (plasmid 1, as defined by the main paper), "I" (plasmid 2), "CH_3" (plasmid 3), "MTC5" (plasmid 4), and "AW" (plasmid 5).

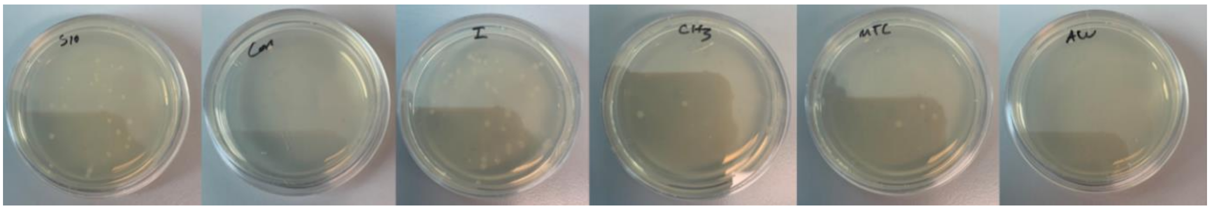

Figure S1. Example ampicillin containing LB agar growth plates for each transformation condition.

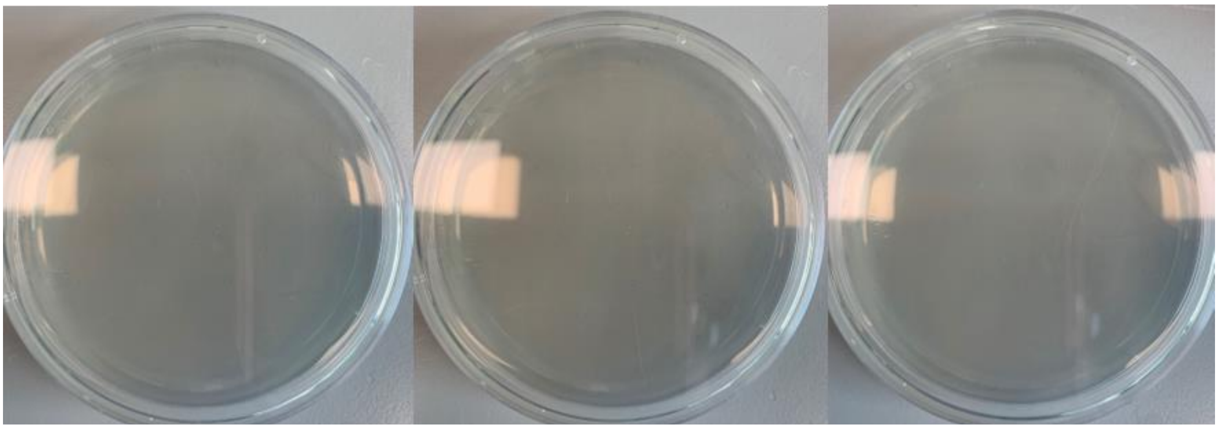

Figure S2. Ampicillin contain plates for 3 separately prepared bacterial samples transformed using AdoHcy azide, imaged after 1, 3, and 7 days (left to right) of growth at 37°C.

The growth assays were performed 3 times. Transformed bacteria containing plasmid 5 were moreover subjected to an extended growth assay over 7 days, figure S2. The results of this assay show that there is no colony formation, indicating that the bacteria cannot grow due to the presence of the ampicillin.

Imaging

Bacteria were sampled from plate colonies and swabbed on a standard glass microscope slide, with a cover slip added. Plate images were taken using a handheld camera. For single cell images, bacterial swabs were diluted in 1 mL of SOC media, with 20 µL pipetted onto a microscope slide and a cover slip added. All slides were imaged using a Nikon Ti2 Eclipse under brightfield and green fluorescence illumination. Internal calibration (1 px = 0.108 µm) was used for all measurements and size calibrations.

Plasmid sizing

Modified and reassembled plasmids were purified using a PCR clean-up kit (GenEluteTM, Sigma-Aldrich). The products were then run on a 1% agarose gel at 75 V for 40 minutes. To 100 mL of 1X Tris-acetate-EDTA buffer (TAE, Tris (hydroxymethyl) aminomethane 1 g, EDTA, tetrasodium < 1 g, Water 98 mL, Disodium EDTA ~1 g, 1,3-Propanediol, 2-amino-2-(hydroxymethyl)-, acetate (salt) ~1 g, Fisher Scientific), 1 g of agarose powder (Sigma-Aldrich) was added. This was microwaved for 2 minutes until the agarose had completely dissolved. The solution was allowed to cool to 50°C then poured into a gel tray with a 15 well comb in place and incubated at room temperature for 30 minutes for the gel to set. To 5 µL of each DNA sample, and DNA ladder (GeneRuler 1 kbp plus DNA Ladder, ThermoFisher), 1 µL of 6X DNA loading buffer (ThermoFisher) was added and homogenised. The gel was then placed into a Mini-Sub Cell GT Cell (BIORAD), and this filled with 1X TAE buffer until the gel was covered. The well comb was then removed and the first well filled with 5 µL of a DNA ladder (GeneRuler 1 kbp DNA Ladder, ThermoFisher). The remaining wells were filled with 5 µL of the DNA samples under analysis or DNA standards. The gel was then placed in 1X Sybr Gold (ThermoFisher) for 45 minutes before imaging in a UV illumination box (BIORAD). The gel images were then analysed using Fiji-ImageJ.

Gel results show that the expected size of the initial plasmids matches the manufacturer specifications and the modifications do not appear to affect the integrity of the plasmids for transformation.

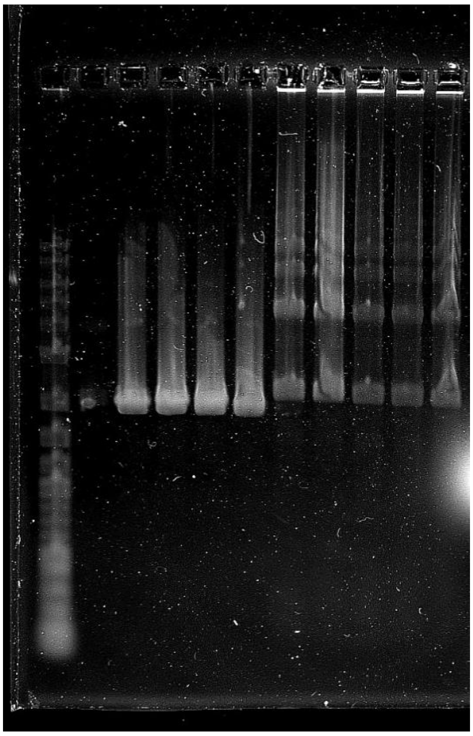

Figure S1. Gel image showing the assembly of modified plasmids (lane 1: gene ruler (1kbp plus, Thermofisher), lane 2: AmpR plasmid 1, lane 3: AmpR plasmid 2, lane 4: AmpR plasmid 3, lane 5: AmpR plasmid 4, lane 6: AmpR plasmid 5, lane 7: eGFP plasmid 1, lane 8: eGFP plasmid 2, lane 9: eGFP plasmid 3, lane 10: eGFP plasmid 4, lane 11: eGFP plasmid 5).

SI2. Sequencing results

Sanger sequencing was performed using Source BioScience, using the primer TCACAAGTATGCTTGGTC (MT 57.5°C). Primers were supplied to the sequencing company at 5 pmol/µL in 5 µL of nuclease free water. Nanopore sequencing was performed using the same company. For both sequencing preparations, plasmids were prepared at 100 ng/µL in nuclease free water.

Sanger sequencing

The raw sequences obtained from Sanger sequencing for AmpR plasmids 1, 2, 4, and 5 are provided in table S2. The text highlighted in yellow indicates the insertion sequence correctly read.

Table S2. AmpR sequencing results from Sanger sequencing

Sample	Sequence
Plasmid 1	NNNNNNNNNNNNNNNNTNNNNNNNNNNNNTGCGATGCGGCCTTGACGGCCTTCCGCCAATTCGCCCTATAGTGAGTCGTATTACGTC<br>GCGCTCACTGGCCGTCGTTTTACAACGTCGTGACTGGGAAAACCCTGGCGTTACCCAACTTAATCGCCTTGCAGCACATCCCCCTTTCG<br>CCAGCTGGCGTAATAGCGAAGAGGCCCGCACCGAAACGCCCTTCCCAACAGTTGCGCAGCCTGAATGGCGAATGGGAGCGCCCTGTA<br>GCGGCCACTCAACCCTATCTCGGTCTATTCTTTTGATTTATAAGGGATTTTGCCGATTTCGGCCTATTGGTTAAAAAATGAGCTGATTTAA<br>CAAAAATTTAACGCGAATTTTAACAAAATATTAACGCTTACAATTTAGGTGGCACTTTTCGGGGAAATGTGCGCGGAACCCCTATTTGTT<br>TATTTTTCTAAATACATTCAAATATGTATCCGCTCATGAGACAATAACCCTGATAAATGCTTCAATAATATTGAAAAAGGAAGAGTATGAGT<br>ATTCAACATTTCCGTGTCGCCCTTATTCCCTTTTTTGCGGCATTTTGCCTTCCTGTTTTTGCTCACCCAGAAACGCTGGTGAAAGTAAAAG<br>ATGCTGAAGATCAGTTGGGTGCACGAGTGGGTTACATCGAACTGGATCTCAACAGCGGTAAGATCCTTGAGAGTTTTCGCCCCGAAGA<br>ACGTTTTCCAATGATGAGCACTTTTAAAGTTCTGCTATGTGGCGCGGTATTATCCCGTATTGACGCCGGGCAAGAGCAACTCGGTCGCC<br>GCATACACTATTCTCAGAATGACTTGGTTGAGTACTCACCAGTCACAGAAAAGCATCTTACGGATGGCATGACAGTAAGAGAATTATGCA<br>GTGCTGCCATAACCATGAGTGATAACACTGCGGCCAACTTACTTCTGACAACGATCGGAGGACCGAAGGAGCTAACCGCTTTTTTGCAC<br>AACATGGGGGATCATGTAACTCGCCTTGATCGTTGGGAACCGGAGCTGAATGAAGCCATACCAAACGACGAGCGTGACACCACGATGC<br>CTGTAGCAATGGCAACAACGTTGCGCAAACTATTAACTGGCGAACTACTTACTCTAGCTTCCCGGCAACAATTAATAGACTGGATGGAG<br>GCGGATAAAGTTGCAGGACCACTTCTGNGCTCGGCCCTTCCGGCTGGCTGGTTTATTGCTGATAANNCTGAAGCCGGTGAGCGGGGT<br>TCTCCGGNNNTCATTGCANNACTGGGGCCAAATGGGAANCCCTCCCGGATTNGNNNTTATNNACCNNACGGGNNAGNNGGNNNC<br>CTTNGGATGAACNNAAAAAAANNNNANNNCT
Plasmid 2	NNNNNNNNNNNNNNNNNNNNNNNNNGNCTGNNTTGGNGTNNNNGCCTGNTGNNACTAAGTNNTTGNANNNNNNNNNANACGC<br>TCNNGNAGGGGNTTGACNNNTCCGAANGNTTTCACCTCGCCCGGTTCTTNCTGAAGCCCCGTGCCTGCACCCCCTCATNGGAGATG<br>NNGNAATACCCAAGAAGGCCATCTCCAATATCCCTTGCCANCAGTTGTTCANCCTGAAGGGCCAATGGGGGNGCCCTGTACCGGCCN<br>CTCCACCCTAGCTCGGCCTATTCTTTTTATTTGTNAGGGACGNNGCTNATTTTGGCCTATTGCTTAANNCTGAGNCTATTTAACANAAAT<br>TTGACGCNANTTNNAACATAATATTAACGCGGNNATTTANGNGTCACTTTTCGGGGAAATGTGCCCTCGAATCGAGTACCGGGAACCC<br>CTATTTGTTTATTTTTCNNACTACATTCAAATATGTATCCGCTCATGAGACGGTAACCCTGATAAATGCTTCCCCACTATTGAAAAAGGAA<br>GAGTATGAGTATACAACATTTCCGNGTCNGCCTTATTCCCTTTTTTGCGGCATTTTGCCTTCGTGTTTTTGCTCACCCNGAAACNNTGGT<br>GANNNTGANAGANNNTGAAAATCAGGGGGGTGCACGAGGGGGCGTACATNNTNACTGCATCTCCCATGCGTAAGATCCTGTGANA<br>GNNTTCTCCCCCCAAGACCNCTTTCCAATGATGAGCACTTTTAAAGNTGTGCTATGTAGCGCTGTNNTAATCNCCNCATTGNNGCNNN<br>CGNAGCAGCGACTCGACCCCCGNATACGCAATT
Plasmid 4	NNNNNNNNNNNNNNNNNNNNNNNNNGGNNNGCGATGCGGCCTTGACGGCCTTCCGCCAATTCGCCCTATAGTGAGTCGTATTACG<br>TCGCGCTCACTGGCCGTCGTTTTACAACGTCGTGACTGGGAAAACCCTGGCGTTACCCAACTTAATCGCCTTGCAGCACATCCCCCTTTC<br>GCCAGCTGGCGTAATAGCGAAGAGGCCCGCACCGAAACGCCCTTCCCAACAGTTGCGCAGCCTGAATGGCGAATGGGAGCGCCCTGT<br>AGCGGCCACTCAACCCTATCTCGGTCTATTCTTTTGATTTATAAGGGATTTTGCCGATTTCGGCCTATTGGTTAAAAAATGAGCTGATTTA<br>ACAAAAATTTAACGCGAATTTTAACAAAATATTAACGCTTACAATTTAGGTGGCACTTCGAATCGAGTACCGTTTCGGGGAAATGTGCGC<br>GGAACCCCTATTTGTTTATTTTTCTAAATACATTCAAATATGTATCCGCTCATGAGACAATAACCCTGATAAATGCTTCAATAATATTGAAAA<br>AGGAAGAGTATGAGTATTCAACATTTCCGTGTCGCCCTTATTCCCTTTTTTGCGGCATTTTGCCTTCCTGTTTTTGCTCACCCAGAAACGC<br>TGGTGAAAGTAAAAGATGCTGAAGATCAGTTGGGTGCACGAGTGGGTTACATCGAACTGGATCTCAACAGCGGTAAGATCCTTGAGA<br>GTTTTCGCCCCGAAGAACGTTTTCCAATGATGAGCACTTTTAAAGTTCTGCTATGTGGCGCGGTATTATCCCGTATTGACGCCGGGCAAG<br>AGCAACTCGGTCGCCGCATACACTATTCTCAGAATGACTTGGTTGAGTACTCACCAGTCACAGAAAAGCATCTTACGGATGGCATGACA<br>GTAAGAGAATTATGCAGTGCTGCCATAACCATGNGTGATAACACTGCGGCCAACTTACTTCTGACAACGATCGGAGGANCGAANGAGC<br>TAACCGCTTTTTTGCACNNCATGGGNGGATCNTGTNACCTCCCCTTGATCGCTCNGAACCGNNACCGACGCACCCNNNCCANACCNC<br>NNCGGNACNCCNNNATCCNTG
Plasmid 5	NNNNNNNNNNNNNNNNNNNNNNNNNNNNNCTGCGATGCGGCCTTGACGGCCTTCCGCCAATTCGCCCTATAGTGAGTCGTATTACGT<br>CGCGCTCACTGGCCGTCGTTTTACAACGTCGTGACTGGGAAAACCCTGGCGTTACCCAACTTAATCGCCTTGCAGCACATCCCCCTTTC<br>GCCAGCTGGCGTAATAGCGAAGAGGCCCGCACCGAAACGCCCTTCCCAACAGTTGCGCAGCCTGAATGGCGAATGGGAGCGCCCTGT<br>AGCGGCCACTCAACCCTATCTCGGTCTATTCTTTTGATTTATAAGGGATTTTGCCGATTTNANCCTATTGGTTAGAAAAAGTGCTGACTTA<br>ACAAAAATGTAANGNNAAATGTAACAAAATATTCCNAACNNACNNNNTCGGNNNNNN

Nanopore sequencing

The raw sequences obtained from Oxford Nanopore sequencing are provided in table S3. Red text indicates the AmpR gene, orange for the AmpR promoter, and the text highlighted in yellow shows the newly added insertion sequence. The blue highlight with the green dotted line in the sequence for AmpR plasmid 5 indicates the point at which the insertion, and part of the promoter sequence, is missing before returning to the original sequence. This site indicates the read termination position shown in the chromatogram in the main manuscript, figure 2. All sequencing positions have been normalized to begin at the AmpR gene for comparability between sample reads.

Table S3. AmpR sequencing results from nanopore sequencing

Sample	Sequence
Plasmid 1	ATGAGTATTCAACATTTCCGTGTCGCCCTTATTCCCTTTTTTGCGGCATTTTGCCTTCCTGTTTTTGCTCACCCAGAAACG<br>CTGGTGAAAGTAAAAGATGCTGAAGATCAGTTGGGTGCACGAGTGGGTTACATCGAACTGGATCTCAACAGCGGTAAG<br>ATCCTTGAGAGTTTTCGCCCCGAAGAACGTTTTCCAATGATGAGCACTTTTAAAGTTCTGCTATGTGGCGCGGTATTATC<br>CCGTATTGACGCCGGGCAAGAGCAACTCGGTCGCCGCATACACTATTCTCAGAATGACTTGGTTGAGTACTCACCAGTC<br>ACAGAAAAGCATCTTACGGATGGCATGACAGTAAGAGAATTATGCAGTGCTGCCATAACCATGAGTGATAACACTGCGG<br>CCAACTTACTTCTGACAACGATCGGAGGACCGAAGGAGCTAACCGCTTTTTTGCACAACATGGGGGATCATGTAACTCG<br>CCTTGATCGTTGGGAACCGGAGCTGAATGAAGCCATACCAAACGACGAGCGTGACACCACGATGCCTGTAGCAATGGC<br>AACAACGTTGCGCAAACTATTAACTGGCGAACTACTTACTCTAGCTTCCCGGCAACAATTAATAGACTGGATGGAGGCG<br>GATAAAGTTGCAGGACCACTTCTGCGCTCGGCCCTTCCGGCTGGCTGGTTTATTGCTGATAAATCTGGAGCCGGTGAGC<br>GTGGTTCTCGCGGTATCATTGCAGCACTGGGGCCAGATGGTAAGCCCTCCCGTATCGTAGTTATCTACACGACGGGGAG<br>TCAGGCAACTATGGATGAACGAAATAGACAGATCGCTGAGATAGGTGCCTCACTGATTAAGCATTGGTAACTGTCAGAC<br>CAAGTTTACTCATATATACTTTAGATTGATTTAAAACTTCATTTTTAATTTAAAAGGATCTAGGTGAAGATCCTTTTTGATA<br>ATCTCATGACCAAAATCCCTTAACGTGAGTTTTCGTTCCACTGAGCGTCAGACCCCGTAGAAAAGATCAAAGGATCTTCT<br>TGAGATCCTTTTTTTCTGCGCGTAATCTGCTGCTTGCAAACAAAAAAACCACCGCTACCAGCGGTGGTTTGTTTGCCGG<br>ATCAAGAGCTACCAACTCTTTTTCCGAAGGTAACTGGCTTCAGCAGAGCGCAGATACCAAATACTGTTCTTCTAGTGTAG<br>CCGTAGTTAGGCCACCACTTCAAGAACTCTGTAGCACCGCCTACATACCTCGCTCTGCTAATCCTGTTACCAGTGGCTGC<br>TGCCAGTGGCGATAAGTCGTGTCTTACCGGGTTGGACTCAAGACGATAGTTACCGGATAAGGCGCAGCGGTCGGGCTG<br>AACGGGGGGTTCGTGCACACAGCCCAGCTTGGAGCGAACGACCTACACCGAACTGAGATACCTACAGCGTGAGCTAT<br>GAGAAAGCGCCACGCTTCCCGAAGGGAGAAAGGCGGACAGGTATCCGGTAAGCGGCAGGGTCGGAACAGGAGAGC<br>GCACGAGGGAGCTTCCAGGGGGAAACGCCTGGTATCTTTATAGTCCTGTCGGGTTTCGCCACCTCTGACTTGAGCGTC<br>GATTTTTGTGATGCTCGTCAGGGGGGCGGAGCCTATGGAAAAACGCCAGCAACGCGGCCTTTTTACGGTTCCTGGCCT<br>TTTGCTGGCCTTTTGCTCATTAGGCACCCCAGGCTTTACCCGAACGACCGAGCGCAGCGAGTCAGTGAGCGAGGAAG<br>CGGAGAGCGCCCAATACGCAAGGAAACAGCTATGACCATGTTAATGCAGCTGGCACGACAGGTTTCCCGACTGGAAA<br>GCGGGCAGTGAGCGGAAGGCCCATGAGGCCCAGATCCAGCAGTCCGACGAATTACCGGAAACTTTATATACAATTAAG<br>AAAATTTACAAATTTGAAGTTGGAACACCCTTCCCTTAGGGCGATTTAGCCACCGCGAGCTGTGTTCCGGAATTCCCGT<br>GAAAGGGGTATTTTACACTGGCTGCGTTGCCGACTTTCTCATACTCGCGATTGCAGAGTAAAGAAATTCCCATGCTAGA<br>CCGTTGCCGGTTCCCATTGTACTTCACGGGGAGCCACAAGCGCCACAGTTCATGACCCTACGCTGAGCAGTGAATAATT<br>ATACCATGGATTAAGCGACCGCCGTGTGCATTTCTCGCGAAACCGTGCTGACTCTTTGGTGTGATAATTCTACAGTCTGA<br>AAGGTCTATTGAGTGTCCTCGCTCTGGGCAACAAAGTATCATCGTCGGGTGAGGTTCGTAGATTTCCGGCGAGATCCTA<br>GATACAAGGTTGCTTCAGGTCTCAAGCCCGCTGATGCTAGAAAACATGTAACAGCGTGACGCACGTTATTTGTAACGTG<br>TCGTTGCAAGAGAGTGTTCGTTGGAAGATCATGCTGGTCCGTCGTTGTTATGCGCTCTGCTGTAACCTGGCTAATGGGT<br>TAAGGTTGTTTGAGATGAGCACACATATGGGCATCCTTCCCAATCCTGTGTCGCGCGCCTTAGCCTGAAAAGACAGGTA<br>GGTGGCGGGATACTACACATTGTTTTCATAATGTTCCCCTTTCATATGACGGCGGGGCATGAATATAGTCGTGTACCATGC<br>CGTACGTTTATGAGATCTCGCATATCTTCACGAAGTGGGTTTCAGTCCCGATTACTCCCCAGGGGTGTAGTGTACGTGAT<br>GCAATAGTAGTGGGATAGTGTCCGATGAACAACCCCAAAACCATTTTAACGGCTATCAGCGAGCACGGGCCGCCAGGT<br>CCTGGCACGAAGGATTGCGAGACTTCCGCTCACAAGTATGCTTGGTCGTGCCAAGTGTCCTCGTCTACGAGCACGGTCC<br>TGCGATGCGGCCTTGACGGCCTTCCGCCAATTCGCCCTATAGTGAGTCGTATTACGTCGCGCTCACTGGCCGTCGTTTTA<br>CAACGTCGTGACTGGGAAAACCCTGGCGTTACCCAACTTAATCGCCTTGCAGCACATCCCCCTTTCGCCAGCTGGCGTA<br>ATAGCGAAGAGGCCCGCACCGAAACGCCCTTCCCAACAGTTGCGCAGCCTGAATGGCGAATGGGAGCGCCCTGTAGC

	GGCCACTCAACCCTATCTCGGTCTATTCTTTTGATTTATAAGGGATTTTGCCGATTTCGGCCTATTGGTTAAAAAATGAGC<br>TGATTTAACAAAAATTTAACGCGAATTTTAACAAAATATTAACGCTTACAATTTAGGTGGCACTTTTCGGGGAAATGTGC<br>GCGGAACCCCTATTTGTTTATTTTTCTAAATACATTCAAATATGTATCCGCTCATGAGACAATAACCCTGATAAATGCTTCA<br>ATAATATTGAAAAAGGAAGAGT
Plasmid 2	ATGAGTATTCAACATTTCCGTGTCGCCCTTATTCCCTTTTTTGCGGCATTTTGCCTTCCTGTTTTTGCTCACCCAGAAACG<br>CTGGTGAAAGTAAAAGATGCTGAAGATCAGTTGGGTGCACGAGTGGGTTACATCGAACTGGATCTCAACAGCGGTAAG<br>ATCCTTGAGAGTTTTCGCCCCGAAGAACGTTTTCCAATGATGAGCACTTTTAAAGTTCTGCTATGTGGCGCGGTATTATC<br>CCGTATTGACGCCGGGCAAGAGCAACTCGGTCGCCGCATACACTATTCTCAGAATGACTTGGTTGAGTACTCACCAGTC<br>ACAGAAAAGCATCTTACGGATGGCATGACAGTAAGAGAATTATGCAGTGCTGCCATAACCATGAGTGATAACACTGCGG<br>CCAACTTACTTCTGACAACGATCGGAGGACCGAAGGAGCTAACCGCTTTTTTGCACAACATGGGGGATCATGTAACTCG<br>CCTTGATCGTTGGGAACCGGAGCTGAATGAAGCCATACCAAACGACGAGCGTGACACCACGATGCCTGTAGCAATGGC<br>AACAACGTTGCGCAAACTATTAACTGGCGAACTACTTACTCTAGCTTCCCGGCAACAATTAATAGACTGGATGGAGGCG<br>GATAAAGTTGCAGGACCACTTCTGCGCTCGGCCCTTCCGGCTGGCTGGTTTATTGCTGATAAATCTGGAGCCGGTGAGC<br>GTGGTTCTCGCGGTATCATTGCAGCACTGGGGCCAGATGGTAAGCCCTCCCGTATCGTAGTTATCTACACGACGGGGAG<br>TCAGGCAACTATGGATGAACGAAATAGACAGATCGCTGAGATAGGTGCCTCACTGATTAAGCATTGGTAACTGTCAGAC<br>CAAGTTTACTCATATATACTTTAGATTGATTTAAAACTTCATTTTTAATTTAAAAGGATCTAGGTGAAGATCCTTTTTGATA<br>ATCTCATGACCAAAATCCCTTAACGTGAGTTTTCGTTCCACTGAGCGTCAGACCCCGTAGAAAAGATCAAAGGATCTTCT<br>TGAGATCCTTTTTTTCTGCGCGTAATCTGCTGCTTGCAAACAAAAAAACCACCGCTACCAGCGGTGGTTTGTTTGCCGG<br>ATCAAGAGCTACCAACTCTTTTTCTATACCATGGATTAAGCGACCGCCGTGTGCATTTCTCGCGAAACCGTGCTGACTCT<br>TTGGTGTGATAATTCTACAGTCTGAAAGGTCTATTGAGTGTCCTCGCTCTGGGCAACAAAGTATCATCGTCGGGTGAGGT<br>TCGTAGATTTCCGGCGAGATCCTAGATACAAGGTTGCTTCAGGTCTCAAGCCCGCTGATGCTAGAAAACATGTAACAGC<br>GTGACGCACGTTATTTGTAACGTGTCGTTGCAAGAGAGTGTTCGTTGGAAGATCATGCTGGTCCGTCGTTGTTATGCGC<br>TCTGCTGTAACCTGGCTAATGGGTTAAGGTTGTTTGAGATGAGCACACATATGGGCATCCTTCCCAATCCTGTGTCGCGC<br>GCCTTAGCCTGAAAAGACAGGTAGGTGGCGGGATACTACACATTGTTTTCATAATGTTCCCCTTTCATATGACGGCGGG<br>GCATGAATATAGTCGTGTACCATGCCGTACGTTTATGAGATCTCGCATATCTTCACGAAGTGGGTTTCAGTCCCGATTACT<br>CCCCAGGGGTGTAGTGTACGTGATGCAATAGTAGTGGGATAGTGTCCGATGAACAACCCCAAAACCATTTTAACGGCTA<br>TCAGCGAGCACGGGCCGCCAGGTCCTGGCACGAAGGATTGCGAGACTTCCGCTCACAAGTATGCTTGGTCGTGCCAA<br>GTGTCCTCGTCTACGAGCACGGTCCTGCGATGCGGCCTTGACGGCCTTCCGCCAATTCGCCCTATAGTGAGTCGTATTAC<br>GTCGCGCTCACTGGCCGTCGTTTTACAACGTCGTGACTGGGAAAACCCTGGCGTTACCCAACTTAATCGCCTTGCAGCA<br>CATCCCCCTTTCGCCAGCTGGCGTAATAGCGAAGAGGCCCGCACCGAAACGCCCTTCCCAACAGTTGCGCAGCCTGAA<br>TGGCGAATGGGAGCGCCCTGTAGCGGCCACTCAACCCTATCTCGGTCTATTCTTTTGATTTATAAGGGATTTTGCCGATT<br>TCGGCCTATTGGTTAAAAAATGAGCTGATTTAACAAAAATTTAACGCGAATTTTAACAAAATATTAACGCTTACAATTTAG<br>GTGGCACTTTCGAATCGAGTACCGCGCGGAACCCCTATTTGTTTATTTTTCTAAATACATTCAAATATGTATCCGCTCATGA<br>GACAATAACCCTGATAAATGCTTCAATAATATTGAAAAAGGAAGAGT
Plasmid 4	ATGAGTATTCAACATTTCCGTGTCGCCCTTATTCCCTTTTTTGCGGCATTTTGCCTTCCTGTTTTTGCTCACCCAGAAACG<br>CTGGTGAAAGTAAAAGATGCTGAAGATCAGTTGGGTGCACGAGTGGGTTACATCGAACTGGATCTCAACAGCGGTAAG<br>ATCCTTGAGAGTTTTCGCCCCGAAGAACGTTTTCCAATGATGAGCACTTTTAAAGTTCTGCTATGTGGCGCGGTATTATC<br>CCGTATTGACGCCGGGCAAGAGCAACTCGGTCGCCGCATACACTATTCTCAGAATGACTTGGTTGAGTACTCACCAGTC<br>ACAGAAAAGCATCTTACGGATGGCATGACAGTAAGAGAATTATGCAGTGCTGCCATAACCATGAGTGATAACACTGCGG<br>CCAACTTACTTCTGACAACGATCGGAGGACCGAAGGAGCTAACCGCTTTTTTGCACAACATGGGGGATCATGTAACTCG<br>CCTTGATCGTTGGGAACCGGAGCTGAATGAAGCCATACCAAACGACGAGCGTGACACCACGATGCCTGTAGCAATGGC<br>AACAACGTTGCGCAAACTATTAACTGGCGAACTACTTACTCTAGCTTCCCGGCAACAATTAATAGACTGGATGGAGGCG<br>GATAAAGTTGCAGGACCACTTCTGCGCTCGGCCCTTCCGGCTGGCTGGTTTATTGCTGATAAATCTGGAGCCGGTGAGC<br>GTGGTTCTCGCGGTATCATTGCAGCACTGGGGCCAGATGGTAAGCCCTCCCGTATCGTAGTTATCTACACGACGGGGAG<br>TCAGGCAACTATGGATGAACGAAATAGACAGATCGCTGAGATAGGTGCCTCACTGATTAAGCATTGGTAACTGTCAGAC<br>CAAGTTTACTCATATATACTTTAGATTGATTTAAAACTTCATTTTTAATTTAAAAGGATCTAGGTGAAGATCCTTTTTGATA<br>ATCTCATGACCAAAATCCCTTAACGTGAGTTTTCGTTCCACTGAGCGTCAGACCCCGTAGAAAAGATCAAAGGATCTTCT<br>TGAGATCCTTTTTTTCTGCGCGTAATCTGCTGCTTGCAAACAAAAAAACCACCGCTACCAGCGGTGGTTTGTTTGCCGG<br>ATCAAGAGCTACCAACTCTTTTTCCGAAGGTAACTGGCTTCAGCAGAGCGCAGATACCAAATACTGTTCTTCTAGTGTAG<br>CCGTAGTTAGGCCACCACTTCAAGAACTCTGTAGCACCGCCTACATACCTCGCTCTGCTAATCCTGTTACCAGTGGCTGC<br>TGCCAGTGGCGATAAGTCGTGTCTTACCGGGTTGGACTCAAGACGATAGTTACCGGATAAGGCGCAGCGGTCGGGCTG<br>AACGGGGGGTTCGTGCACACAATTCTACAGTCTGAAAGGTCTATTGAGTGTCCTCGCTCTGGGCAACAAAGTATCATCG<br>TCGGGTGAGGTTCGTAGATTTCCGGCGAGATCCTAGATACAAGGTTGCTTCAGGTCTCAAGCCCGCTGATGCTAGAAAA<br>CATGTAACAGCGTGACGCACGTTATTTGTAACGTGTCGTTGCAAGAGAGTGTTCGTTGGAAGATCATGCTGGTCCGTCG<br>TTGTTATGCGCTCTGCTGTAACCTGGCTAATGGGTTAAGGTTGTTTGAGATGAGCACACATATGGGCATCCTTCCCAATC<br>CTGTGTCGCGCGCCTTAGCCTGAAAAGACAGGTAGGTGGCGGGATACTACACATTGTTTTCATAATGTTCCCCTTTCATA<br>TGACGGCGGGGCATGAATATAGTCGTGTACCATGCCGTACGTTTATGAGATCTCGCATATCTTCACGAAGTGGGTTTCAG<br>TCCCGATTACTCCCCAGGGGTGTAGTGTACGTGATGCAATAGTAGTGGGATAGTGTCCGATGAACAACCCCAAAACCAT<br>TTTAACGGCTATCAGCGAGCACGGGCCGCCAGGTCCTGGCACGAAGGATTGCGAGACTTCCGCTCACAAGTATGCTTG<br>GTCGTGCCAAGTGTCCTCGTCTACGAGCACGGTCCTGCGATGCGGCCTTGACGGCCTTCCGCCAATTCGCCCTATAGTG<br>AGTCGTATTACGTCGCGCTCACTGGCCGTCGTTTTACAACGTCGTGACTGGGAAAACCCTGGCGTTACCCAACTTAATC<br>GCCTTGCAGCACATCCCCCTTTCGCCAGCTGGCGTAATAGCGAAGAGGCCCGCACCGAAACGCCCTTCCCAACAGTTG<br>CGCAGCCTGAATGGCGAATGGGAGCGCCCTGTAGCGGCCACTCAACCCTATCTCGGTCTATTCTTTTGATTTATAAGGG

	ATTTTGCCGATTTCGGCCTATTGGTTAAAAAATGAGCTGATTTAACAAAAATTTAACGCGAATTTTAACAAAATATTAACG<br>CTTACAATTTAGGTGGCACTCGAATCGAGTACCGGCGCGGAACCCCTATTTGTTTATTTTTCTAAATACATTCAAATATGT<br>ATCCGCTCATGAGACAATAACCCTGATAAATGCTTCAATAATATTGAAAAAGGAAGAGT
Plasmid 5	TTACCAATGCTTAATCAGTGAGGCACCTATCTCAGCGATCTGTCTATTTCGTTCATCCATAGTTGCCTGACTCCCCGTCGTG<br>TAGATAACTACGATACGGGAGGGCTTACCATCTGGCCCCAGTGCTGCAATGATACCGCGAGAACCACGCTCACCGGCTC<br>CAGATTTATCAGCAATAAACCAGCCAGCCGGAAGGGCCGAGCGCAGAAGTGGTCCTGCAACTTTATCCGCCTCCATCC<br>AGTCTATTAATTGTTGCCGGGAAGCTAGAGTAAGTAGTTCGCCAGTTAATAGTTTGCGCAACGTTGTTGCCATTGCTACA<br>GGCATCGTGGTGTCACGCTCGTCGTTTGGTATGGCTTCATTCAGCTCCGGTTCCCAACGATCAAGGCGAGTTACATGATC<br>CCCCATGTTGTGCAAAAAAGCGGTTAGCTCCTTCGGTCCTCCGATCGTTGTCAGAAGTAAGTTGGCCGCAGTGTTATCA<br>CTCATGGTTATGGCAGCACTGCATAATTCTCTTACTGTCATGCCATCCGTAAGATGCTTTTCTGTGACTGGTGAGTACTCA<br>ACCAAGTCATTCTGAGAATAGTGTATGCGGCGACCGAGTTGCTCTTGCCCGGCGTCAATACGGGATAATACCGCGCCAC<br>ATAGCAGAACTTTAAAAGTGCTCATCATTGGAAAACGTTCTTCGGGGCGAAAACTCTCAAGGATCTTACCGCTGTTGAG<br>ATCCAGTTCGATGTAACCCACTCGTGCACCCAACTGATCTTCAGCATCTTTTACTTTCACCAGCGTTTCTGGGTGAGCAA<br>AAACAGGAAGGCAAAATGCCGCAAAAAAGGGAATAAGGGCGACACGGAAATGTTGAATACTCATACTCTTCCTTTTTC<br>AATATTATTGAAGCATTTATCAGGGTTATTGTCTCATGAGCGGATACATATTTGAATGTATTTAGAAAAATAAACAAATAGG<br>GGTTCCGCGCACATTTCCCCGAAAAGTGCCACCTAAATTGTAAGCGTTAATATTTTGTTAAAATTCGCGTTAAATTTTTG<br>TTAAATCAGCTCATTTTTTAACCAATAGGCCGAAATCGGCAAAATCCCTTATAAATCAAAAGAATAGACCGAGATAGGGT<br>TGAGTGGCCGCTACAGGGCGCTCCCATTCGCCATTCAGGCTGCGCAACTGTTGGGAAGGGCGTTTCGGTGCGGGCCT<br>CTTCGCTATTACGCCAGCTGGCGAAAGGGGGATGTGCTGCAAGGCGATTAAGTTGGGTAACGCCAGGGTTTTCCCAGT<br>CACGACGTTGTAAAACGACGGCCAGTGAGCGCGACGTAATACGACTCACTATAGGGCGAATTGGCGGAAGGCCGTCA<br>AGGCCGCATCGCAGCCAGCTGCATTAACATGGTCATAGCTGTTTCCTTGCGTATTGGGCGCTCTCCGCTTCCTCGCTCAC<br>TGACTCGCTGCGCTCGGTCGTTCGGGTAAAGCCTGGGGTGCCTAATGAGCAAAAGGCCAGCAAAAGGCCAGGAACCG<br>TAAAAAGGCCGCGTTGCTGGCGTTTTTCCATAGGCTCCGCCCCCCTGACGAGCATCACAAAAATCGACGCTCAAGTCA<br>GAGGTGGCGAAACCCGACAGGACTATAAAGATACCAGGCGTTTCCCCCTGGAAGCTCCCTCGTGCGCTCTCCTGTTCC<br>GACCCTGCCGCTTACCGGATACCTGTCCGCCTTTCTCCCTTCGGGAAGCGTGGCGCTTTCTCATAGCTCACGCTGTAGGT<br>ATCTCAGTTCGGTGTAGGTCGTTCGCTCCAAGCTGGGCTGTGTGCACGAACCCCCCGTTCAGCCCGACCGCTGCGCCTT<br>ATCCGGTAACTATCGTCTTGAGTCCAACCCGGTAAGACACGACTTATCGCCACTGGCAGCAGCCACTGGTAACAGGATT<br>AGCAGAGCGAGGTATGTAGGCGGTGCTACAGAGTTCTTGAAGTGGTGGCCTAACTACGGCTACACTAGAAGAACAGTA<br>TTTGGTATCTGCGCTCTGCTGAAGCCAGTTACCTTCGGAAAAAGAGTTGGTAGCTCTTGATCCGGCAAACAAACCACCG<br>CTGGTAGCGGTGGTTTTTTTGTTTGCAAGCAGCAGATTACGCGCAGAAAAAAAGGATCTCAAGAAGATCCTTTGATCTT<br>TTCTACGGGGTCTGACGCTCAGTGGAACGAAAACTCACGTTAAGGGATTTTGGTCATGAGATTATCAAAAAGGATCTTC<br>ACCTAGATCCTTTTAAATTAAAAATGAAGTTTTAAATCAATCTAAAGTATATATGAGTAAACTTGGTCTGACAG

Dual gene containing plasmid characterisation

To determine the after-effect of the modification on the pCSDEST2-EGFP plasmid *in vivo*, BL21 cells were grown in liquid LB broth, and plasmids extracted using a Miniprep kit (Qiagen). The wash solutions were collected for further analysis to determine if there was any genomic integration of the plasmid DNA due to the disruption caused by the modification. Both plasmid and remaining DNA were subjected to PCR amplification (Q5 Hot Start, NEB, 98°C denaturing-10 seconds, 52°C annealing- 30 seconds, 72°C extension- 30 seconds, 30 cycles) for eGFP and AmpR, and the resulting products were run on a gel, as shown in figure S4. The primer sequences are shown below. As we discuss in the main paper, the AmpR gene is active and the eGFP gene is not, therefore primers were designed to avoid the promoter regions to determine if the genes were still present or had been excised.

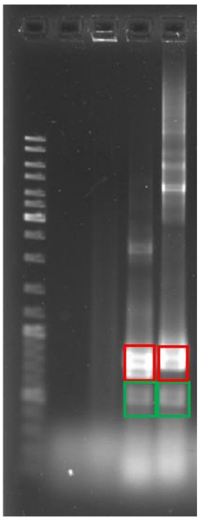

Figure S2. 1% agarose gel electrophoresis results of PCR amplified AmpR (red) and eGFP (green) genes from extracted, unmodified and modified, eGFP plasmids (lane 1: gene ruler (1kbp plus, Thermofisher), lane 2: genomic DNA, modified plasmid, lane 3: genomic DNA, unmodified plasmid, lane 4: modified plasmid amplification, lane:5 unmodified plasmid amplification).

<u>AmpR primers</u>

5’- TTA CCA ATG CTT AAT CAG TGA GGC ACC -3’

5’- CAC CCA GAA ACG CTG GTG AAA -3’

<u>eGFP primers</u>

5’- ATG GTG AGC AAG GGC GAG GA -3’

5’- CTT GTA CAG CTC GTC CAT GCC -3’

From the results of the amplification, it is possible to conclude that there is no genomic integration due to the lack of bands. The bands generated from the PCR amplified, post-growth, extracted plasmids, show that both AmpR and eGFP genes are present. Interestingly, this also showed that in the unmodified plasmids, higher weight bands than the modified plasmid were observed, indicative of restriction. The decreased yield from plasmid extraction, without the colony numbers being affected, were; modified plasmids at 25ng/ µL, and unmodified plasmids at 251ng/ µL, also indicates a decrease

in copy number. In combination, these results suggested removal or a portion of the DNA containing the modified site. To determine the effect of the modification during the cell cycle, the extracted plasmid was sequenced using ONT, the results of which are shown in table S4. Red text indicates the AmpR gene, green for the eGFP promoter, and the text highlighted in purple shows the sequence missing from the modified post extraction sample.

Table S4. Plasmid 6 sequencing results from nanopore sequencing

Sample	Sequence
Unmodified post-growth eGFP plasmid	GCCATTCAGGCTGCGCAACTGTTGGGAAGGGCGATCGGTGCGGGCCTCTTCGCTATTACGCCAG<br>TCGACCGCCAATTCAATATGGCGTATATGGACTCATGCCAATTCAATATGGTGGATCTGGACCTGTG<br>CCAATTCAATATGGCGTATATGGACTCGTGCCAATTCAATATGGTGGATCTGGACCCCAGCCAATTC<br>AATATGGCGGACTTGGCACCATGCCAATTCAATATGGCGGACCTGGCACTGTGCCAACTGGGGAG<br>GGGTCTACTTGGCACGGTGCCAAGTTTGAGGAGGGGTCTTGGCCCTGTGCCAAGTCCGCCATAT<br>TGAATTGGCATGGTGCCAATAATGGCGGCCATATTGGCTATATGCCAGGATCAATATATAGGCAATA<br>TCCAATATGGCCCTATGCCAATATGGCTATTGGCCAGGTTCAATACTATGTATTGGCCCTATGCCATA<br>TAGTATTCCATATATGGGTTTTCCTATTGACGTAGATAGCCCCTCCCAATGGGCGGTCCCATATACCA<br>TATATGGGGCTTCCTAATACCGCCCATAGCCACTCCCCCATTGACGTCAATGGTCTCTATATATGGTC<br>TTTCCTATTGACGTCATATGGGCGGTCCTATTGACGTATATGGCGCCTCCCCCATTGACGTCAATTAC<br>GGTAAATGGCCCGCCTGGCTCAATGCCCATTGACGTCAATAGGACCACCCACCATTGACGTCAAT<br>GGGATGGCTCATTGCCCATTCATATCCGTTCTCACGCCCCCTATTGACGTCAATGACGGTAAATGGC<br>CCACTTGGCAGTACATCAATATCTATTAATAGTAACTTGGCAAGTACATTACTATTGGAAGTACGCC<br>AGGGTACATTGGCAGTACTCCCATTGACGTCAATGGCGGTAAATGGCCCGCGATGGCTGCCAAGT<br>ACATCCCCATTGACGTCAATGGGGAGGGGCAATGACGCAAATGGGCGTTCCATTGACGTAAATG<br>GGCGGTAGGCGTGCCTAATGGGAGGTCTATATAAGCAATGCTCGTTTAGGGAACCGCCATTCTGC<br>CTGGGGACGTCGGAGCAAGCTTGATTTAGGTGACACTATAGAATACAAGCTACTTGTTCTTTTTGC<br>AGGATCCCATCGATTCGAATTCAAGGATCAACAAGTTTGTACAAAAAAGCAGGCTGCCACCATGG<br>TGAGCAAGGGCGAGGAGCTGTTCACCGGGGTGGTGCCCATCCTGGTCGAGCTGGACGGCGACG<br>TAAACGGCCACAAGTTCAGCGTGTCCGGCGAGGGCGAGGGCGATGCCACCTACGGCAAGCTGA<br>CCCTGAAGTTCATCTGCACCACCGGCAAGCTGCCCGTGCCCTGGCCCACCCTCGTGACCACCCTG<br>ACCTACGGCGTGCAGTGCTTCAGCCGCTACCCCGACCACATGAAGCAGCACGACTTCTTCAAGTC<br>CGCCATGCCCGAAGGCTACGTCCAGGAGCGCACCATCTTCTTCAAGGACGACGGCAACTACAAG<br>ACCCGCGCCGAGGTGAAGTTCGAGGGCGACACCCTGGTGAACCGCATCGAGCTGAAGGGCATC<br>GACTTCAAGGAGGACGGCAACATCCTGGGGCACAAGCTGGAGTACAACTACAACAGCCACAAC<br>GTCTATATCATGGCCGACAAGCAGAAGAACGGCATCAAGGTGAACTTCAAGATCCGCCACAACAT<br>CGAGGACGGCAGCGTGCAGCTCGCCGACCACTACCAGCAGAACACCCCCATCGGCGACGGCCC<br>CGTGCTGCTGCCCGACAACCACTACCTGAGCACCCAGTCCGCCCTGAGCAAAGACCCCAACGAG<br>AAGCGCGATCACATGGTCCTGCTGGAGTTCGTGACCGCCGCCGGGATCACTCTCGGCATGGACG<br>AGCTGTACAAGCACCCAGCTTTCTTGTACAAAGTGGTGGCGAAAACGTACGACTATCTGTTCAAA<br>CTGCTGCTCATCGGAGACAGCGGTGTGGGCAAGACCTGTCTGCTGTTTCGATTCAGCGAGGACG<br>CCTTCAACACCACTTTCATCTCCACCATAGGAATTGACTTCAAAATCAGAACAATTGAGCTGGATG<br>GGAAAAAGATCAAGCTTCAAATCTGGGATACGGCGGGACAGGAGAGGTTCAGAACCATCACCA<br>CTGCATATTACAGAGGAGCAATGGGAATTATGCTGGTGTATGATATCACAAATGAGAAATCATTTG<br>ACAACATCAAGAACTGGATCAGGAACATTGAGGAGCATGCATCGTCAGATGTGGAGCGAATGAT<br>ATTGGGTAACAGGTGTGACATGAACGACAAGAGACAAGTTTCAAAAGAAAGAGGAGAGAAGCT<br>AGCTATTGATTACGGAATCAAGTTCTTGGAAACCAGTGCAAAATCTAGCACAAATGTTGAAGAGG

	CATTTGTCACTCTTGCTAGAGACATCATGACGAGATTAAACAGGAAAATGAATGAGAATAACCCTT<br>CAGGGGGAGGAGGAGGAGGAGCAGTAAAAATCACAGAGAGTCGATCCAAGAAGCCAAGCTTC<br>TTCCGCTGCATTCTGCTCTAACCCAGCCAACTTTATTATACATAGTTGCACTCGAGCCTCTAGAACT<br>ATAGTGAGTCGTATTACGTAGATCCAGACATGATAAGATACATTGATGAGTTTGGACAAACCACAA<br>CTAGAATGCAGTGAAAAAAATGCTTTATTTGTGAAATTTGTGATGCTATTGCTTTATTTGTAACCAT<br>TATAAGCTGCAATAAACAAGTTAACAACAACAATTGCATTCATTTTATGTTTCAGGTTCAGGGGGA<br>GGTGTGGGAGGTTTTTTAATTCGCGGCCGCGGCGCCAATGCATTGGGCCCGGTACCCAGCTTTT<br>GTTCCCTTTAGTGAGGGTTAATTGCGCGCTTGGCGTAATCATGGTCATAGCTGTTTCCTGTGTGAA<br>ATTGTTATCCGCTCACAATTCCACACAACATACGAGCCGGGAGCATAAAGTGTAAAGCCTGGGGT<br>GCCTAATGAGTGAGCTAACTCACATTAATTGCGTTGCGCTCACTGCCCGCTTTCCAGTCGGGAAAC<br>CTGTCGTGCCAGCTGCATTAATGAATCGGCCAACGCGCGGGGAGAGGCGGTTTGCGTATTGGGC<br>GCTCTTCCGCTTCCTCGCTCACTGACTCGCTGCGCTCGGTCGTTCGGCTGCGGCGAGCGGTATCA<br>GCTCACTCAAAGGCGGTAATACGGTTATCCACAGAATCAGGGGATAACGCAGGAAAGAACATGT<br>GAGCAAAAGGCCAGCAAAAGGCCAGGAACCGTAAAAAGGCCGCGTTGCTGGCGTTTTTCCATA<br>GGCTCCGCCCCCCTGACGAGCATCACAAAAATCGACGCTCAAGTCAGAGGTGGCGAAACCCGAC<br>AGGACTATAAAGATACCAGGCGTTTCCCCCTGGAAGCTCCCTCGTGCGCTCTCCTGTTCCGACCCT<br>GCCGCTTACCGGATACCTGTCCGCCTTTCTCCCTTCGGGAAGCGTGGCGCTTTCTCATAGCTCACG<br>CTGTAGGTATCTCAGTTCGGTGTAGGTCGTTCGCTCCAAGCTGGGCTGTGTGCACGAACCCCCCG<br>TTCAGCCCGACCGCTGCGCCTTATCCGGTAACTATCGTCTTGAGTCCAACCCGGTAAGACACGACT<br>TATCGCCACTGGCAGCAGCCACTGGTAACAGGATTAGCAGAGCGAGGTATGTAGGCGGTGCTAC<br>AGAGTTCTTGAAGTGGTGGCCTAACTACGGCTACACTAGAAGAACAGTATTTGGTATCTGCGCTC<br>TGCTGAAGCCAGTTACCTTCGGAAAAAGAGTTGGTAGCTCTTGATCCGGCAAACAAACCACCGC<br>TGGTAGCGGTGGTTTTTTTGTTTGCAAGCAGCAGATTACGCGCAGAAAAAAAGGATCTCAAGAA<br>GATCCTTTGATCTTTTCTACGGGGTCTGACGCTCAGTGGAACGAAAACTCACGTTAAGGGATTTT<br>GGTCATGAGATTATCAAAAAGGATCTTCACCTAGATCCTTTTAAATTAAAAATGAAGTTTTAAATCA<br>ATCTAAAGTATATATGAGTAAACTTGGTCTGACAGTTACCAATGCTTAATCAGTGAGGCACCTATCT<br>CAGCGATCTGTCTATTTCGTTCATCCATAGTTGCCTGACTCCCCGTCGTGTAGATAACTACGATACG<br>GGAGGGCTTACCATCTGGCCCCAGTGCTGCAATGATACCGCGAGACCCACGCTCACCGGCTCCA<br>GATTTATCAGCAATAAACCAGCCAGCCGGAAGGGCCGAGCGCAGAAGTGGTCCTGCAACTTTAT<br>CCGCCTCCATCCAGTCTATTAATTGTTGCCGGGAAGCTAGAGTAAGTAGTTCGCCAGTTAATAGTT<br>TGCGCAACGTTGTTGCCATTGCTACAGGCATCGTGGTGTCACGCTCGTCGTTTGGTATGGCTTCAT<br>TCAGCTCCGGTTCCCAACGATCAAGGCGAGTTACATGATCCCCCATGTTGTGCAAAAAAGCGGTT<br>AGCTCCTTCGGTCCTCCGATCGTTGTCAGAAGTAAGTTGGCCGCAGTGTTATCACTCATGGTTATG<br>GCAGCACTGCATAATTCTCTTACTGTCATGCCATCCGTAAGATGCTTTTCTGTGACTGGTGAGTACT<br>CAACCAAGTCATTCTGAGAATAGTGTATGCGGCGACCGAGTTGCTCTTGCCCGGCGTCAATACGG<br>GATAATACCGCGCCACATAGCAGAACTTTAAAAGTGCTCATCATTGGAAAACGTTCTTCGGGGCG<br>AAAACTCTCAAGGATCTTACCGCTGTTGAGATCCAGTTCGATGTAACCCACTCGTGCACCCAACTG<br>ATCTTCAGCATCTTTTACTTTCACCAGCGTTTCTGGGTGAGCAAAAACAGGAAGGCAAAATGCCG<br>CAAAAAAGGGAATAAGGGCGACACGGAAATGTTGAATACTCATACTCTTCCTTTTTCAATATTATT<br>GAAGCATTTATCAGGGTTATTGTCTCATGAGCGGATACATATTTGAATGTATTTAGAAAAATAAACA<br>AATAGGGGTTCCGCGCACATTTCCCCGAAAAGTGCCACCTAAATTGTAAGCGTTAATATTTTGTTA<br>AAATTCGCGTTAAATTTTTGTTAAATCAGCTCATTTTTTAACCAATAGGCCGAAATCGGCAAAATCC<br>CTTATAAATCAAAAGAATAGACCGAGATAGGGTTGAGTGTTGTTCCAGTTTGGAACAAGAGTCCA<br>CTATTAAAGAACGTGGACTCCAACGTCAAAGGGCGAAAAACCGTCTATCAGGGCGATGGCCCAC<br>TACGTGAACCATCACCCTAATCAAGTTTTTTGGGGTCGAGGTGCCGTAAAGCACTAAATCGGAAC<br>CCTAAAGGGAGCCCCCGATTTAGAGCTTGACGGGGAAAGCCGGCGAACGTGGCGAGAAAGGA<br>AGGGAAGAAAGCGAAAGGAGCGGGCGCTAGGGCGCTGGCAAGTGTAGCGGTCACGCTGCGCG<br>TAACCACCACACCCGCCGCGCTTAATGCGCCGCTACAGGGCGCGTCCCATTC

Modified post-growth eGFP plasmid	ACGCGCCCTGTAGCGGCGCATTAAGCGCGGCGGGTGTGGTGGTTACGCGCAGCGTGACCGCTAC<br>ACTTGCCAGCGCCCTAGCGCCCGCTCCTTTCGCTTTCTTCCCTTCCTTTCTCGCCACGTTCGCCGG<br>CTTTCCCCGTCAAGCTCTAAATCGGGGGCTCCCTTTAGGGTTCCGATTTAGTGCTTTACGGCACCT<br>CGACCCCAAAAAACTTGATTAGGGTGATGGTTCACGTAGTGGGCCATCGCCCTGATAGACGGTTT<br>TTCGCCCTTTGACGTTGGAGTCCACGTTCTTTAATAGTGGACTCTTGTTCCAAACTGGAACAACAC<br>TCAACCCTATCTCGGTCTATTCTTTTGATTTATAAGGGATTTTGCCGATTTCGGCCTATTGGTTAAAA<br>AATGAGCTGATTTAACAAAAATTTAACGCGAATTTTAACAAAATATTAACGCTTACAATTTAGGTG<br>GCACTTTTCGGGGAAATGTGCGCGGAACCCCTATTTGTTTATTTTTCTAAATACATTCAAATATGTA<br>TCCGCTCATGAGACAATAACCCTGATAAATGCTTCAATAATATTGAAAAAGGAAGAGTATGAGTAT<br>TCAACATTTCCGTGTCGCCCTTATTCCCTTTTTTGCGGCATTTTGCCTTCCTGTTTTTGCTCACCCAG<br>AAACGCTGGTGAAAGTAAAAGATGCTGAAGATCAGTTGGGTGCACGAGTGGGTTACATCGAACT<br>GGATCTCAACAGCGGTAAGATCCTTGAGAGTTTTCGCCCCGAAGAACGTTTTCCAATGATGAGCA<br>CTTTTAAAGTTCTGCTATGTGGCGCGGTATTATCCCGTATTGACGCCGGGCAAGAGCAACTCGGTC<br>GCCGCATACACTATTCTCAGAATGACTTGGTTGAGTACTCACCAGTCACAGAAAAGCATCTTACGG<br>ATGGCATGACAGTAAGAGAATTATGCAGTGCTGCCATAACCATGAGTGATAACACTGCGGCCAAC<br>TTACTTCTGACAACGATCGGAGGACCGAAGGAGCTAACCGCTTTTTTGCACAACATGGGGGATC<br>ATGTAACTCGCCTTGATCGTTGGGAACCGGAGCTGAATGAAGCCATACCAAACGACGAGCGTGA<br>CACCACGATGCCTGTAGCAATGGCAACAACGTTGCGCAAACTATTAACTGGCGAACTACTTACTCT<br>AGCTTCCCGGCAACAATTAATAGACTGGATGGAGGCGGATAAAGTTGCAGGACCACTTCTGCGCT<br>CGGCCCTTCCGGCTGGCTGGTTTATTGCTGATAAATCTGGAGCCGGTGAGCGTGGGTCTCGCGGT<br>ATCATTGCAGCACTGGGGCCAGATGGTAAGCCCTCCCGTATCGTAGTTATCTACACGACGGGGAG<br>TCAGGCAACTATGGATGAACGAAATAGACAGATCGCTGAGATAGGTGCCTCACTGATTAAGCATT<br>GGTAACTGTCAGACCAAGTTTACTCATATATACTTTAGATTGATTTAAAACTTCATTTTTAATTTAAA<br>AGGATCTAGGTGAAGATCCTTTTTGATAATCTCATGACCAAAATCCCTTAACGTGAGTTTTCGTTCC<br>ACTGAGCGTCAGACCCCGTAGAAAAGATCAAAGGATCTTCTTGAGATCCTTTTTTTCTGCGCGTA<br>ATCTGCTGCTTGCAAACAAAAAAACCACCGCTACCAGCGGTGGTTTGTTTGCCGGATCAAGAGCT<br>ACCAACTCTTTTTCCGAAGGTAACTGGCTTCAGCAGAGCGCAGATACCAAATACTGTTCTTCTAGT<br>GTAGCCGTAGTTAGGCCACCACTTCAAGAACTCTGTAGCACCGCCTACATACCTCGCTCTGCTAAT<br>CCTGTTACCAGTGGCTGCTGCCAGTGGCGATAAGTCGTGTCTTACCGGGTTGGACTCAAGACGAT<br>AGTTACCGGATAAGGCGCAGCGGTCGGGCTGAACGGGGGGTTCGTGCACACAGCCCAGCTTGG<br>AGCGAACGACCTACACCGAACTGAGATACCTACAGCGTGAGCTATGAGAAAGCGCCACGCTTCC<br>CGAAGGGAGAAAGGCGGACAGGTATCCGGTAAGCGGCAGGGTCGGAACAGGAGAGCGCACG<br>AGGGAGCTTCCAGGGGGAAACGCCTGGTATCTTTATAGTCCTGTCGGGTTTCGCCACCTCTGACT<br>TGAGCGTCGATTTTTGTGATGCTCGTCAGGGGGGCGGAGCCTATGGAAAAACGCCAGCAACGCG<br>GCCTTTTTACGGTTCCTGGCCTTTTGCTGGCCTTTTGCTCACATGTTCTTTCCTGCGTTATCCCCTG<br>ATTCTGTGGATAACCGTATTACCGCCTTTGAGTGAGCTGATACCGCTCGCCGCAGCCGAACGACC<br>GAGCGCAGCGAGTCAGTGAGCGAGGAAGCGGAAGAGCGCCCAATACGCAAACCGCCTCTCCCC<br>GCGCGTTGGCCGATTCATTAATGCAGGATCTCGATCCCGCGAAATTAATACGACTCACTATAGGGA<br>GACCACAACGGTTTCCCTCTAGAAATAATTTTGTTTAACTTTAAGAAGGAGATATACATATGCGGG<br>GTTCTCATCATCATCATCATCATGGTATGGCTAGCATGACTGGTGGACAGCAAATGGGTCGGGATC<br>TGTACGACGATGACGATAAGGATCCCATGGTGAGCAAGGGCGAGGAGCTGTTCACCGGGGTGG<br>TGCCCATCCTGGTCGAGCTGGACGGCGACGTAAACGGCCACAAGTTCAGCGTGTCCGGCGAGG<br>GCGAGGGCGATGCCACCTACGGCAAGCTGACCCTGAAGTTCATCTGCACCACCGGCAAGCTGCC<br>CGTGCCCTGGCCCACCCTCGTGACCACCCTGACCTACGGCGTGCAGTGCTTCAGCCGCTACCCCG<br>ACCACATGAAGCAGCACGACTTCTTCAAGTCCGCCATGCCCGAAGGCTACGTCCAGGAGCGCAC<br>CATCTTCTTCAAGGACGACGGCAACTACAAGACCCGCGCCGAGGTGAAGTTCGAGGGCGACACC<br>CTGGTGAACCGCATCGAGCTGAAGGGCATCGACTTCAAGGAGGACGGCAACATCCTGGGGCAC<br>AAGCTGGAGTACAACTACAACAGCCACAACGTCTATATCATGGCCGACAAGCAGAAGAACGGCA<br>TCAAGGTGAACTTCAAGATCCGCCACAACATCGAGGACGGCAGCGTGCAGCTCGCCGACCACTA<br>CCAGCAGAACACCCCCATCGGCGACGGCCCCGTGCTGCTGCCCGACAACCACTACCTGAGCACC<br>CAGTCCGCCCTGAGCAAAGACCCCAACGAGAAGCGCGATCACATGGTCCTGCTGGAGTTCGTGA<br>CCGCCGCCGGGATCACTCTCGGCATGGACGAGCTGTACAAGTAAGAATTCGAAGCTTGATCCGG

	CTGCTAACAAAGCCCGAAAGGAAGCTGAGTTGGCTGCTGCCACCGCTGAGCAATAACTAGCATA ACCCCTTGGGGCCTCTAAACGGGTCTTGAGGGGTTTTTTGCTGAAAGGAGGAACTATATCCGGAT CTGGCGTAATAGCGAAGAGGCCCGCACCGATCGCCCTTCCCAACAGTTGCGCAGCCTGAATGGC GAATGGG

From the results of the sequencing, we hypothesize that, during replication the bacteria employes its restriction enzymes to remove the section that it is unable to replicate, then repairs the damaged region, reforming a smaller plasmid. This would be an energy intensive process and therefore it is likely that the bacteria prioritize the modification of fewer plasmids, ejecting or not copying excess plasmids, reducing the copy number as the cells divide, and therefore the yield obtained from extraction.

Whilst the mechanism by which this process occurs is currently unknown, we suggest that the AdoHcy azide triggers the host excision and repair pathways. This would cause the removal of the modification site and surrounding DNA then reseal the gaps left to produce a new, smaller plasmid missing vital sequences to create an active gene.

SI3. Ligation insertion sequence stability

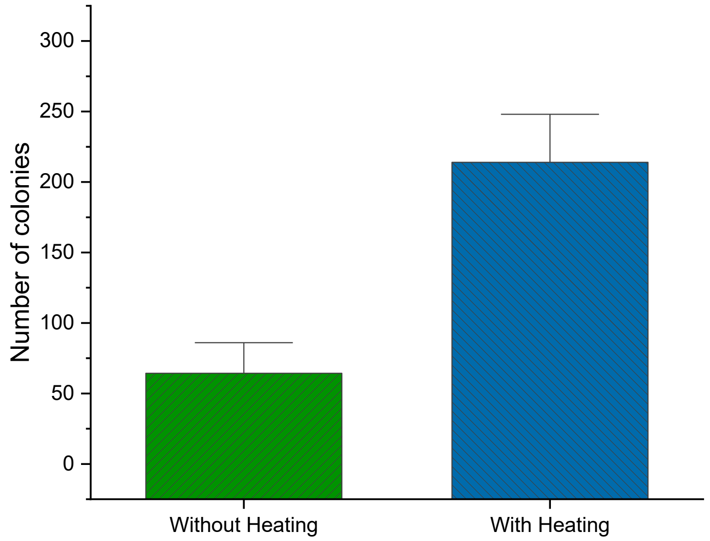

Figure S3. Bar chart showing the difference in colony count when the ligation insertion plasmid is prepared without and with a 50°C heating step respectively.

Initially, the insertion sequence was ligated into the plasmid directly after the initial digestion step. However, it was noticed that the colony count was significantly lower than the CH_3 or MTC5 modified plasmids in the ampicillin containing plates. As the sequences are identical, we thought this was unusual, therefore the ligation plasmids were re-prepared with an additional heating step at 50°C, post ligation. We found that the additional heating step assisted with stabilising the formation of the final plasmid, improving the overall transformation, and bringing the colony count to similar levels to those observed in the other modified plasmids, as shown in figure S5.

SI4. Statistical analysis

For statistical analysis, the presence of an antibiotic was selected as one factor (two levels) and the plasmid modification as the second factor (five levels) for a two-way ANOVA comparison. All colony counts are provided in table S5. Normality testing was performed using the Shapiro-Wilk test, table S6. Different plasmids were analysed individually to determine the effect of plasmid modification on bacterial growth. Results from this analysis show significant differences in the colony numbers observed for the native bacteria, bacteria with plasmids 1-4, and with plasmid 5. A summary table of the two-way ANOVA results is shown in table S7, with the individual condition statistics shown in tables S8-S11. Statistical analysis of the colony counts for the bacteria transformed with the eGFP plasmid is shown in table S12.

Table S5. Colony counts for all experiments (after 24 hours)

Antibiotic presence on plate	Bacterial strain	Plasmid	Colony count (N=1)	Colony count (N=2)	Colony count (N=3)
No AMP	DH5α	No plasmid	225	306	285
		1	210	191	220
		2	188	170	146
		3	196	164	202
		4	201	198	222
		5	180	155	194
AMP		No plasmid	0	0	0
		1	219	198	251
		2	52	86	55
		3	201	164	250
		4	186	158	205
		5	0	0	0
No AMP	BL21	No plasmid	322	347	306
		1	210	284	224
		2	240	263	265
		3	257	209	239
		4	211	244	236
		5	228	257	225
AMP		No plasmid	0	0	0
		1	267	233	216
		2	280	228	259
		3	275	242	255
		4	198	271	251
		5	0	0	0
No Kan	DH5α	No plasmid	301	294	277
		1	286	246	236
		2	276	239	255
		3	202	243	194
		4	258	271	226
		5	231	205	251
Kan		No plasmid	0	0	0
		1	235	231	207
		2	260	279	242
		3	204	279	258
		4	214	211	202
		5	0	0	0
No Kan	BL21	No plasmid	350	311	302
		1	290	255	228
		2	275	240	211
		3	240	229	207
		4	267	253	239
		5	271	244	221
Kan		No plasmid	0	0	0
		1	280	222	206
		2	261	236	252
		3	255	201	230
		4	237	197	215
		5	0	0	0
AMP	DH5α	6a	287	224	296
		No plasmid	0	0	0
		6b	253	254	211
AMP	BL21	6a	268	251	286
		No plasmid	0	0	0
		6b	210	246	208

a*: All colony counts were 0.

Table S6. Normality testing of all bacterial conditions using the Shapiro-Wilk test

Bacterial condition	Plasmid condition	DF	Statistic	p-value	Decision at level(5%)
DH5α No Ampicllin	No transformation	3	0.92827	0.48216	Can't reject normality
	Control	3	0.96889	0.66139	Can't reject normality
	Ligation insert	3	0.99324	0.84283	Can't reject normality
	CH3	3	0.86502	0.28148	Can't reject normality
	MTC5	3	0.84211	0.21956	Can't reject normality
	AdoHcy-azide	3	0.97417	0.6917	Can't reject normality
DH5α Ampicllin	No transformation	3	--	--	a*
	Control	3	0.98584	0.77223	Can't reject normality
	Ligation insert	3	0.99933	0.95064	Can't reject normality
	CH3	3	0.99355	0.84647	Can't reject normality
	MTC5	3	0.98792	0.78971	Can't reject normality
	AdoHcy-azide	3	--	--	a*
BL21 No Kanamycin	No transformation	3	0.88479	0.33861	Can't reject normality
	Control	3	0.99448	0.85798	Can't reject normality
	Ligation insert	3	0.99708	0.89673	Can't reject normality
	CH3	3	0.96429	0.63689	Can't reject normality
	MTC5	3	1	1	Can't reject normality
	AdoHcy-azide	3	0.99787	0.91185	Can't reject normality
BL21 Kanamycin	No transformation	3	--	--	a*
	Control	3	0.90303	0.39523	Can't reject normality
	Ligation insert	3	0.97453	0.6939	Can't reject normality
	CH3	3	0.99817	0.91837	Can't reject normality
	MTC5	3	0.99668	0.88986	Can't reject normality
	AdoHcy-azide	3	--	--	a*
BL21 No Ampicllin	No transformation	3	0.98419	0.75924	Can't reject normality
	Control	3	0.88589	0.34191	Can't reject normality
	Ligation insert	3	0.80959	0.13759	Can't reject normality
	CH3	3	0.97959	0.72623	Can't reject normality
	MTC5	3	0.91873	0.44788	Can't reject normality
	AdoHcy-azide	3	0.81964	0.16229	Can't reject normality
BL21 Ampicllin	No transformation	3	--	--	a*
	Control	3	0.96429	0.63689	Can't reject normality
	Ligation insert	3	0.98782	0.78881	Can't reject normality
	CH3	3	0.98522	0.76726	Can't reject normality
	MTC5	3	0.93623	0.51241	Can't reject normality
	AdoHcy-azide	3	--	--	a*
DH5α No Kanamycin	No transformation	3	0.9453	0.54913	Can't reject normality
	Control	3	0.89286	0.36311	Can't reject normality
	Ligation insert	3	0.99395	0.85129	Can't reject normality
	CH3	3	0.86867	0.29175	Can't reject normality
	MTC5	3	0.94391	0.54334	Can't reject normality
	AdoHcy-azide	3	0.99436	0.85645	Can't reject normality
DH5α Kanamycin	No transformation	3	--	--	a*
	Control	3	0.85465	0.25297	Can't reject normality
	Ligation insert	3	0.99976	0.9702	Can't reject normality
	CH3	3	0.93938	0.52488	Can't reject normality
	MTC5	3	0.92308	0.46326	Can't reject normality
	AdoHcy-azide	3	--	--	a*
DH5α GFP plasmid, Amicillin plates	Control	3	0.84211	0.21956	Can't reject normality
	No transformation	3	--	--	a*
	AdoHcy-Azide	3	0.76743	0.03891	Reject normality
BL21 GFP plasmid, Amicillin plates	Control	3	0.99973	0.9685	Can't reject normality
	No transformation	3	--	--	a*
	AdoHcy-Azide	3	0.78936	0.08934	Can't reject normality

Table S7. Summary table of the statistically significant results from the colony growth assay

DH5α Ampicillin resistance gene					BL21 Ampicillin resistance gene				
Antibiotic	Modification	Antibiotic	Modification	Prob	Antibiotic	Modification	Antibiotic	Modification	Prob
AMP	Control	AMP	No transformation	3.18E-08	AMP	No transformation	NoAMP	No transformation	2.83E-07
AMP	AdoHcy	AMP	Control	3.18E-08	AMP	AdoHcy	NoAMP	No transformation	2.83E-07
AMP	Ligation insert	AMP	No transformation	3.96E-08	AMP	No transformation	NoAMP	Control	3.42E-06
AMP	AdoHcy	AMP	Ligation insert	3.96E-08	AMP	AdoHcy	NoAMP	Control	3.42E-06
AMP	No transformation	NoAMP	Control	5.15E-08	AMP	No transformation	NoAMP	MTC5	5.59E-06
AMP	No transformation	NoAMP	MTC5	5.15E-08	AMP	AdoHcy	NoAMP	MTC5	5.59E-06
AMP	AdoHcy	NoAMP	Control	5.15E-08	AMP	No transformation	NoAMP	Ligation insert	6.18E-06
AMP	AdoHcy	NoAMP	MTC5	5.15E-08	AMP	No transformation	NoAMP	AdoHcy	6.18E-06
AMP	CH3	AMP	No transformation	5.69E-08	AMP	AdoHcy	NoAMP	Ligation insert	6.18E-06
AMP	AdoHcy	AMP	CH3	5.69E-08	AMP	AdoHcy	NoAMP	AdoHcy	6.18E-06
AMP	No transformation	NoAMP	CH3	1.34E-07	AMP	Control	AMP	No transformation	8.09E-06
AMP	AdoHcy	NoAMP	CH3	1.34E-07	AMP	AdoHcy	AMP	Control	8.09E-06
AMP	MTC5	AMP	No transformation	2.30E-07	AMP	Ligation insert	AMP	No transformation	8.99E-06
AMP	AdoHcy	AMP	MTC5	2.30E-07	AMP	AdoHcy	AMP	Ligation insert	8.99E-06
AMP	No transformation	NoAMP	No transformation	2.66E-07	AMP	No transformation	NoAMP	CH3	1.63E-05
AMP	AdoHcy	NoAMP	No transformation	2.66E-07	AMP	AdoHcy	NoAMP	CH3	1.63E-05
AMP	No transformation	NoAMP	AdoHcy	4.90E-07	AMP	CH3	AMP	No transformation	2.20E-05
AMP	AdoHcy	NoAMP	AdoHcy	4.90E-07	AMP	AdoHcy	AMP	CH3	2.20E-05
AMP	No transformation	NoAMP	Ligation insert	1.23E-06	AMP	MTC5	AMP	No transformation	3.70E-05
AMP	AdoHcy	NoAMP	Ligation insert	1.23E-06	AMP	AdoHcy	AMP	MTC5	3.70E-05
NoAMP	Ligation insert	NoAMP	No transformation	0.00178	AMP	MTC5	NoAMP	No transformation	0.01388
NoAMP	AdoHcy	NoAMP	No transformation	0.00469	AMP	CH3	NoAMP	No transformation	0.02884
AMP	MTC5	NoAMP	No transformation	0.01008	NoAMP	CH3	NoAMP	No transformation	0.04447
NoAMP	CH3	NoAMP	No transformation	0.01642					
DH5α Kanamycin resistance gene					BL21 Kanamycin resistance gene				
Antibiotic	Modification	Antibiotic	Modification	Prob	Antibiotic	Modification	Antibiotic	Modification	Prob
Kan	No transformation	NoAMP	No transformation	4.14E-06	Kan	No transformation	NoKan	No transformation	4.20E-07
Kan	AdoHcy	NoAMP	No transformation	4.14E-06	Kan	AdoHcy	NoKan	No transformation	4.20E-07
Kan	CH3	AMP	No transformation	6.12E-05	Kan	Ligation insert	Kan	No transformation	1.73E-06
Kan	AdoHcy	AMP	CH3	6.12E-05	Kan	AdoHcy	Kan	Ligation insert	1.73E-06
Kan	Ligation insert	AMP	No transformation	7.34E-05	Kan	No transformation	NoKan	Control	1.97E-06
Kan	AdoHcy	AMP	Ligation insert	7.34E-05	Kan	AdoHcy	NoKan	Control	1.97E-06
Kan	No transformation	NoAMP	Ligation insert	8.11E-05	Kan	No transformation	NoKan	MTC5	3.54E-06
Kan	AdoHcy	NoAMP	Ligation insert	8.11E-05	Kan	AdoHcy	NoKan	MTC5	3.54E-06
Kan	Control	AMP	No transformation	8.61E-05	Kan	No transformation	NoKan	Ligation insert	4.11E-06
Kan	AdoHcy	AMP	Control	8.61E-05	Kan	AdoHcy	NoKan	Ligation insert	4.11E-06
Kan	No transformation	NoAMP	Control	9.73E-05	Kan	CH3	Kan	No transformation	5.30E-06
Kan	AdoHcy	NoAMP	Control	9.73E-05	Kan	AdoHcy	Kan	CH3	5.30E-06
Kan	No transformation	NoAMP	AdoHcy	1.17E-04	Kan	Control	Kan	No transformation	7.74E-06
Kan	AdoHcy	NoAMP	AdoHcy	1.17E-04	Kan	AdoHcy	Kan	Control	7.74E-06
Kan	MTC5	AMP	No transformation	1.64E-04	Kan	No transformation	NoKan	CH3	1.25E-05
Kan	AdoHcy	AMP	MTC5	1.64E-04	Kan	AdoHcy	NoKan	CH3	1.25E-05
Kan	No transformation	NoAMP	CH3	1.75E-04	Kan	No transformation	NoKan	AdoHcy	1.54E-05
Kan	AdoHcy	NoAMP	CH3	1.75E-04	Kan	AdoHcy	NoKan	AdoHcy	1.54E-05
Kan	No transformation	NoAMP	MTC5	2.21E-04	Kan	MTC5	Kan	No transformation	2.02E-05
Kan	AdoHcy	NoAMP	MTC5	2.21E-04	Kan	AdoHcy	Kan	MTC5	2.02E-05

Table S8. Two-way ANOVA results of DH5α with and without modified plasmids on control and ampicillin plates (statistically significant results highlighted in red).

Factor A	Factor B	Factor A	Factor B	MeanDiff	SEM	q Value	Prob	Alpha	Sig	LCL	UCL
NoAMP	Control	NoAMP	No transformat	-65	20.63169	4.45547	0.1272	0.05	0	-139.39	9.39027
NoAMP	Ligation ins	NoAMP	No transformat	-104	20.63169	7.12875	0.00178	0.05	1	-178.39	-29.6097
NoAMP	Ligation ins	NoAMP	Control	-39	20.63169	2.67328	0.75395	0.05	0	-113.39	35.39027
NoAMP	CH3	NoAMP	No transformat	-84.6667	20.63169	5.80354	0.01642	0.05	1	-159.057	-10.2764
NoAMP	CH3	NoAMP	Control	-19.6667	20.63169	1.34807	0.99746	0.05	0	-94.0569	54.72361
NoAMP	CH3	NoAMP	Ligation insert	19.33333	20.63169	1.32522	0.99781	0.05	0	-55.0569	93.72361
NoAMP	MTC5	NoAMP	No transformat	-65	20.63169	4.45547	0.1272	0.05	0	-139.39	9.39027
NoAMP	MTC5	NoAMP	Control	0	20.63169	0	1	0.05	0	-74.3903	74.39027
NoAMP	MTC5	NoAMP	Ligation insert	39	20.63169	2.67328	0.75395	0.05	0	-35.3903	113.3903
NoAMP	MTC5	NoAMP	CH3	19.66667	20.63169	1.34807	0.99746	0.05	0	-54.7236	94.05694
NoAMP	AdoHcy	NoAMP	No transformat	-95.6667	20.63169	6.55754	0.00469	0.05	1	-170.057	-21.2764
NoAMP	AdoHcy	NoAMP	Control	-30.6667	20.63169	2.10207	0.93047	0.05	0	-105.057	43.72361
NoAMP	AdoHcy	NoAMP	Ligation insert	8.33333	20.63169	0.57121	1	0.05	0	-66.0569	82.72361
NoAMP	AdoHcy	NoAMP	CH3	-11	20.63169	0.754	0.99999	0.05	0	-85.3903	63.39027
NoAMP	AdoHcy	NoAMP	MTC5	-30.6667	20.63169	2.10207	0.93047	0.05	0	-105.057	43.72361
AMP	No transfor	NoAMP	No transformat	-272	20.63169	18.64443	2.66E-07	0.05	1	-346.39	-197.61
AMP	No transfor	NoAMP	Control	-207	20.63169	14.18896	5.15E-08	0.05	1	-281.39	-132.61
AMP	No transfor	NoAMP	Ligation insert	-168	20.63169	11.51568	1.23E-06	0.05	1	-242.39	-93.6097
AMP	No transfor	NoAMP	CH3	-187.333	20.63169	12.84089	1.34E-07	0.05	1	-261.724	-112.943
AMP	No transfor	NoAMP	MTC5	-207	20.63169	14.18896	5.15E-08	0.05	1	-281.39	-132.61
AMP	No transfor	NoAMP	AdoHcy	-176.333	20.63169	12.08689	4.90E-07	0.05	1	-250.724	-101.943
AMP	Control	NoAMP	No transformat	-49.3333	20.63169	3.38159	0.44713	0.05	0	-123.724	25.05694
AMP	Control	NoAMP	Control	15.66667	20.63169	1.07388	0.99967	0.05	0	-58.7236	90.05694
AMP	Control	NoAMP	Ligation insert	54.66667	20.63169	3.74716	0.30754	0.05	0	-19.7236	129.0569
AMP	Control	NoAMP	CH3	35.33333	20.63169	2.42195	0.84578	0.05	0	-39.0569	109.7236
AMP	Control	NoAMP	MTC5	15.66667	20.63169	1.07388	0.99967	0.05	0	-58.7236	90.05694
AMP	Control	NoAMP	AdoHcy	46.33333	20.63169	3.17595	0.53579	0.05	0	-28.0569	120.7236
AMP	Control	NoAMP	No transformat	222.6667	20.63169	15.26284	3.18E-08	0.05	1	148.2764	297.0569
AMP	Ligation ins	NoAMP	No transformat	-58	20.63169	3.97565	0.23611	0.05	0	-132.39	16.39027
AMP	Ligation ins	NoAMP	Control	7	20.63169	0.47982	1	0.05	0	-67.3903	81.39027
AMP	Ligation ins	NoAMP	Ligation insert	46	20.63169	3.1531	0.54589	0.05	0	-28.3903	120.3903
AMP	Ligation ins	NoAMP	CH3	26.66667	20.63169	1.82789	0.97231	0.05	0	-47.7236	101.0569
AMP	Ligation ins	NoAMP	MTC5	7	20.63169	0.47982	1	0.05	0	-67.3903	81.39027
AMP	Ligation ins	NoAMP	AdoHcy	37.66667	20.63169	2.58189	0.7895	0.05	0	-36.7236	112.0569
AMP	Ligation ins	AMP	No transformat	214	20.63169	14.66878	3.96E-08	0.05	1	139.6097	288.3903
AMP	Ligation ins	AMP	Control	-8.66667	20.63169	0.59406	1	0.05	0	-83.0569	65.72361
AMP	CH3	NoAMP	No transformat	-67	20.63169	4.59256	0.10517	0.05	0	-141.39	7.39027
AMP	CH3	NoAMP	Control	-2	20.63169	0.13709	1	0.05	0	-76.3903	72.39027
AMP	CH3	NoAMP	Ligation insert	37	20.63169	2.53619	0.80641	0.05	0	-37.3903	111.3903
AMP	CH3	NoAMP	CH3	17.66667	20.63169	1.21097	0.99901	0.05	0	-56.7236	92.05694
AMP	CH3	NoAMP	MTC5	-2	20.63169	0.13709	1	0.05	0	-76.3903	72.39027
AMP	CH3	NoAMP	AdoHcy	28.66667	20.63169	1.96498	0.9547	0.05	0	-45.7236	103.0569
AMP	CH3	AMP	No transformat	205	20.63169	14.05187	5.69E-08	0.05	1	130.6097	279.3903
AMP	CH3	AMP	Control	-17.6667	20.63169	1.21097	0.99901	0.05	0	-92.0569	56.72361
AMP	CH3	AMP	Ligation insert	-9	20.63169	0.61691	1	0.05	0	-83.3903	65.39027
AMP	MTC5	NoAMP	No transformat	-89	20.63169	6.10057	0.01008	0.05	1	-163.39	-14.6097
AMP	MTC5	NoAMP	Control	-24	20.63169	1.6451	0.98722	0.05	0	-98.3903	50.39027
AMP	MTC5	NoAMP	Ligation insert	15	20.63169	1.02819	0.99978	0.05	0	-59.3903	89.39027
AMP	MTC5	NoAMP	CH3	-4.33333	20.63169	0.29703	1	0.05	0	-78.7236	70.05694
AMP	MTC5	NoAMP	MTC5	-24	20.63169	1.6451	0.98722	0.05	0	-98.3903	50.39027
AMP	MTC5	NoAMP	AdoHcy	6.66667	20.63169	0.45697	1	0.05	0	-67.7236	81.05694
AMP	MTC5	AMP	No transformat	183	20.63169	12.54386	2.30E-07	0.05	1	108.6097	257.3903
AMP	MTC5	AMP	Control	-39.6667	20.63169	2.71898	0.7354	0.05	0	-114.057	34.72361
AMP	MTC5	AMP	Ligation insert	-31	20.63169	2.12492	0.92574	0.05	0	-105.39	43.39027
AMP	MTC5	AMP	CH3	-22	20.63169	1.50801	0.99355	0.05	0	-96.3903	52.39027
AMP	AdoHcy	NoAMP	No transformat	-272	20.63169	18.64443	2.66E-07	0.05	1	-346.39	-197.61
AMP	AdoHcy	NoAMP	Control	-207	20.63169	14.18896	5.15E-08	0.05	1	-281.39	-132.61
AMP	AdoHcy	NoAMP	Ligation insert	-168	20.63169	11.51568	1.23E-06	0.05	1	-242.39	-93.6097
AMP	AdoHcy	NoAMP	CH3	-187.333	20.63169	12.84089	1.34E-07	0.05	1	-261.724	-112.943
AMP	AdoHcy	NoAMP	MTC5	-207	20.63169	14.18896	5.15E-08	0.05	1	-281.39	-132.61
AMP	AdoHcy	NoAMP	AdoHcy	-176.333	20.63169	12.08689	4.90E-07	0.05	1	-250.724	-101.943
AMP	AdoHcy	AMP	No transformat	0	20.63169	0	1	0.05	0	-74.3903	74.39027
AMP	AdoHcy	AMP	Control	-222.667	20.63169	15.26284	3.18E-08	0.05	1	-297.057	-148.276
AMP	AdoHcy	AMP	Ligation insert	-214	20.63169	14.66878	3.96E-08	0.05	1	-288.39	-139.61
AMP	AdoHcy	AMP	CH3	-205	20.63169	14.05187	5.69E-08	0.05	1	-279.39	-130.61
AMP	AdoHcy	AMP	MTC5	-183	20.63169	12.54386	2.30E-07	0.05	1	-257.39	-108.61

Table S9. Two-way ANOVA results of DH5α with and without modified plasmids on control and kanamycin plates (statistically significant results highlighted in red).

Antibiotic	Modificatio	Antibiotic	Modificatio	MeanDiff	SEM	q Value	Prob	Alpha	Sig	LCL	UCL
NoKan	Control	NoKan	No transfor	-31.5	21.94216	2.03023	0.93336	0.05	0	-118.614	55.61375
NoKan	Ligation ins	NoKan	No transfor	-40	21.94216	2.57808	0.78116	0.05	0	-127.114	47.11375
NoKan	Ligation ins	NoKan	Control	-8.5	21.94216	0.54784	1	0.05	0	-95.6138	78.61375
NoKan	CH3	NoKan	No transfor	-75	21.94216	4.83389	0.1178	0.05	0	-162.114	12.11375
NoKan	CH3	NoKan	Control	-43.5	21.94216	2.80366	0.69721	0.05	0	-130.614	43.61375
NoKan	CH3	NoKan	Ligation ins	-35	21.94216	2.25582	0.8819	0.05	0	-122.114	52.11375
NoKan	MTC5	NoKan	No transfor	-33	21.94216	2.12691	0.91342	0.05	0	-120.114	54.11375
NoKan	MTC5	NoKan	Control	-1.5	21.94216	0.09668	1	0.05	0	-88.6138	85.61375
NoKan	MTC5	NoKan	Ligation ins	7	21.94216	0.45116	1	0.05	0	-80.1138	94.11375
NoKan	MTC5	NoKan	CH3	42	21.94216	2.70698	0.73411	0.05	0	-45.1138	129.1138
NoKan	AdoHcy	NoKan	No transfor	-79.5	21.94216	5.12393	0.08603	0.05	0	-166.614	7.61375
NoKan	AdoHcy	NoKan	Control	-48	21.94216	3.09369	0.58292	0.05	0	-135.114	39.11375
NoKan	AdoHcy	NoKan	Ligation ins	-39.5	21.94216	2.54585	0.79243	0.05	0	-126.614	47.61375
NoKan	AdoHcy	NoKan	CH3	-4.5	21.94216	0.29003	1	0.05	0	-91.6138	82.61375
NoKan	AdoHcy	NoKan	MTC5	-46.5	21.94216	2.99701	0.62118	0.05	0	-133.614	40.61375
Kan	No transfor	NoKan	No transfor	-297.5	21.94216	19.17444	4.20E-07	0.05	1	-384.614	-210.386
Kan	No transfor	NoKan	Control	-266	21.94216	17.1442	1.97E-06	0.05	1	-353.114	-178.886
Kan	No transfor	NoKan	Ligation ins	-257.5	21.94216	16.59636	4.11E-06	0.05	1	-344.614	-170.386
Kan	No transfor	NoKan	CH3	-222.5	21.94216	14.34055	1.25E-05	0.05	1	-309.614	-135.386
Kan	No transfor	NoKan	MTC5	-264.5	21.94216	17.04752	3.54E-06	0.05	1	-351.614	-177.386
Kan	No transfor	NoKan	AdoHcy	-218	21.94216	14.05051	1.54E-05	0.05	1	-305.114	-130.886
Kan	Control	NoKan	No transfor	-64.5	21.94216	4.15715	0.23672	0.05	0	-151.614	22.61375
Kan	Control	NoKan	Control	-33	21.94216	2.12691	0.91342	0.05	0	-120.114	54.11375
Kan	Control	NoKan	Ligation ins	-24.5	21.94216	1.57907	0.98718	0.05	0	-111.614	62.61375
Kan	Control	NoKan	CH3	10.5	21.94216	0.67674	0.99999	0.05	0	-76.6138	97.61375
Kan	Control	NoKan	MTC5	-31.5	21.94216	2.03023	0.93336	0.05	0	-118.614	55.61375
Kan	Control	NoKan	AdoHcy	15	21.94216	0.96678	0.99979	0.05	0	-72.1138	102.1138
Kan	Control	Kan	No transfor	233	21.94216	15.01729	7.74E-06	0.05	1	145.8863	320.1138
Kan	Ligation ins	NoKan	No transfor	-28	21.94216	1.80465	0.96772	0.05	0	-115.114	59.11375
Kan	Ligation ins	NoKan	Control	3.5	21.94216	0.22558	1	0.05	0	-83.6138	90.61375
Kan	Ligation ins	NoKan	Ligation ins	12	21.94216	0.77342	0.99998	0.05	0	-75.1138	99.11375
Kan	Ligation ins	NoKan	CH3	47	21.94216	3.02924	0.60841	0.05	0	-40.1138	134.1138
Kan	Ligation ins	NoKan	MTC5	5	21.94216	0.32226	1	0.05	0	-82.1138	92.11375
Kan	Ligation ins	NoKan	AdoHcy	51.5	21.94216	3.31927	0.49558	0.05	0	-35.6138	138.6138
Kan	Ligation ins	Kan	No transfor	269.5	21.94216	17.36978	1.73E-06	0.05	1	182.3863	356.6138
Kan	Ligation ins	Kan	Control	36.5	21.94216	2.35249	0.85474	0.05	0	-50.6138	123.6138
Kan	CH3	NoKan	No transfor	-56	21.94216	3.60931	0.39206	0.05	0	-143.114	31.11375
Kan	CH3	NoKan	Control	-24.5	21.94216	1.57907	0.98718	0.05	0	-111.614	62.61375
Kan	CH3	NoKan	Ligation ins	-16	21.94216	1.03123	0.99962	0.05	0	-103.114	71.11375
Kan	CH3	NoKan	CH3	19	21.94216	1.22459	0.99829	0.05	0	-68.1138	106.1138
Kan	CH3	NoKan	MTC5	-23	21.94216	1.48239	0.99199	0.05	0	-110.114	64.11375
Kan	CH3	NoKan	AdoHcy	23.5	21.94216	1.51462	0.99058	0.05	0	-63.6138	110.6138
Kan	CH3	Kan	No transfor	241.5	21.94216	15.56513	5.30E-06	0.05	1	154.3863	328.6138
Kan	CH3	Kan	Control	8.5	21.94216	0.54784	1	0.05	0	-78.6138	95.61375
Kan	CH3	Kan	Ligation ins	-28	21.94216	1.80465	0.96772	0.05	0	-115.114	59.11375
Kan	MTC5	NoKan	No transfor	-85	21.94216	5.47841	0.05818	0.05	0	-172.114	2.11375
Kan	MTC5	NoKan	Control	-53.5	21.94216	3.44818	0.44804	0.05	0	-140.614	33.61375
Kan	MTC5	NoKan	Ligation ins	-45	21.94216	2.90033	0.65942	0.05	0	-132.114	42.11375
Kan	MTC5	NoKan	CH3	-10	21.94216	0.64452	1	0.05	0	-97.1138	77.11375
Kan	MTC5	NoKan	MTC5	-52	21.94216	3.3515	0.48349	0.05	0	-139.114	35.11375
Kan	MTC5	NoKan	AdoHcy	-5.5	21.94216	0.35449	1	0.05	0	-92.6138	81.61375
Kan	MTC5	Kan	No transfor	212.5	21.94216	13.69603	2.02E-05	0.05	1	125.3863	299.6138
Kan	MTC5	Kan	Control	-20.5	21.94216	1.32126	0.99678	0.05	0	-107.614	66.61375
Kan	MTC5	Kan	Ligation ins	-57	21.94216	3.67376	0.37089	0.05	0	-144.114	30.11375
Kan	MTC5	Kan	CH3	-29	21.94216	1.8691	0.95957	0.05	0	-116.114	58.11375
Kan	AdoHcy	NoKan	No transfor	-297.5	21.94216	19.17444	4.20E-07	0.05	1	-384.614	-210.386
Kan	AdoHcy	NoKan	Control	-266	21.94216	17.1442	1.97E-06	0.05	1	-353.114	-178.886
Kan	AdoHcy	NoKan	Ligation ins	-257.5	21.94216	16.59636	4.11E-06	0.05	1	-344.614	-170.386
Kan	AdoHcy	NoKan	CH3	-222.5	21.94216	14.34055	1.25E-05	0.05	1	-309.614	-135.386
Kan	AdoHcy	NoKan	MTC5	-264.5	21.94216	17.04752	3.54E-06	0.05	1	-351.614	-177.386
Kan	AdoHcy	NoKan	AdoHcy	-218	21.94216	14.05051	1.54E-05	0.05	1	-305.114	-130.886
Kan	AdoHcy	Kan	No transfor	0	21.94216	0	1	0.05	0	-87.1138	87.11375
Kan	AdoHcy	Kan	Control	-233	21.94216	15.01729	7.74E-06	0.05	1	-320.114	-145.886
Kan	AdoHcy	Kan	Ligation ins	-269.5	21.94216	17.36978	1.73E-06	0.05	1	-356.614	-182.386
Kan	AdoHcy	Kan	CH3	-241.5	21.94216	15.56513	5.30E-06	0.05	1	-328.614	-154.386
Kan	AdoHcy	Kan	MTC5	-212.5	21.94216	13.69603	2.02E-05	0.05	1	-299.614	-125.386

Table S10. Two-way ANOVA results of BL21 with and without modified plasmids on control and ampicillin plates (statistically significant results highlighted in red).

Antibiotic	Modificatio	Antibiotic	Modificatio	MeanDiff	SEM	q Value	Prob	Alpha	Sig	LCL	UCL
NoAMP	Control	NoAMP	No transfor	-87.5	29.68024	4.16923	0.23393	0.05	0	-205.335	30.33512
NoAMP	Ligation ins	NoAMP	No transfor	-83	29.68024	3.95481	0.2875	0.05	0	-200.835	34.83512
NoAMP	Ligation ins	NoAMP	Control	4.5	29.68024	0.21442	1	0.05	0	-113.335	122.3351
NoAMP	CH3	NoAMP	No transfor	-101.5	29.68024	4.8363	0.11749	0.05	0	-219.335	16.33512
NoAMP	CH3	NoAMP	Control	-14	29.68024	0.66708	0.99999	0.05	0	-131.835	103.8351
NoAMP	CH3	NoAMP	Ligation ins	-18.5	29.68024	0.88149	0.99991	0.05	0	-136.335	99.33512
NoAMP	MTC5	NoAMP	No transfor	-107	29.68024	5.09837	0.08847	0.05	0	-224.835	10.83512
NoAMP	MTC5	NoAMP	Control	-19.5	29.68024	0.92914	0.99986	0.05	0	-137.335	98.33512
NoAMP	MTC5	NoAMP	Ligation ins	-24	29.68024	1.14356	0.99905	0.05	0	-141.835	93.83512
NoAMP	MTC5	NoAMP	CH3	-5.5	29.68024	0.26207	1	0.05	0	-123.335	112.3351
NoAMP	AdoHcy	NoAMP	No transfor	-92	29.68024	4.38365	0.18875	0.05	0	-209.835	25.83512
NoAMP	AdoHcy	NoAMP	Control	-4.5	29.68024	0.21442	1	0.05	0	-122.335	113.3351
NoAMP	AdoHcy	NoAMP	Ligation ins	-9	29.68024	0.42883	1	0.05	0	-126.835	108.8351
NoAMP	AdoHcy	NoAMP	CH3	9.5	29.68024	0.45266	1	0.05	0	-108.335	127.3351
NoAMP	AdoHcy	NoAMP	MTC5	15	29.68024	0.71472	0.99999	0.05	0	-102.835	132.8351
AMP	No transfor	NoAMP	No transfor	-334.5	29.68024	15.93836	4.14E-06	0.05	1	-452.335	-216.665
AMP	No transfor	NoAMP	Control	-247	29.68024	11.76913	9.73E-05	0.05	1	-364.835	-129.165
AMP	No transfor	NoAMP	Ligation ins	-251.5	29.68024	11.98355	8.11E-05	0.05	1	-369.335	-133.665
AMP	No transfor	NoAMP	CH3	-233	29.68024	11.10206	1.75E-04	0.05	1	-350.835	-115.165
AMP	No transfor	NoAMP	MTC5	-227.5	29.68024	10.83999	2.21E-04	0.05	1	-345.335	-109.665
AMP	No transfor	NoAMP	AdoHcy	-242.5	29.68024	11.55472	1.17E-04	0.05	1	-360.335	-124.665
AMP	Control	NoAMP	No transfor	-84.5	29.68024	4.02628	0.26868	0.05	0	-202.335	33.33512
AMP	Control	NoAMP	Control	3	29.68024	0.14294	1	0.05	0	-114.835	120.8351
AMP	Control	NoAMP	Ligation ins	-1.5	29.68024	0.07147	1	0.05	0	-119.335	116.3351
AMP	Control	NoAMP	CH3	17	29.68024	0.81002	0.99996	0.05	0	-100.835	134.8351
AMP	Control	NoAMP	MTC5	22.5	29.68024	1.07209	0.99947	0.05	0	-95.3351	140.3351
AMP	Control	NoAMP	AdoHcy	7.5	29.68024	0.35736	1	0.05	0	-110.335	125.3351
AMP	Control	AMP	No transfor	250	29.68024	11.91208	8.61E-05	0.05	1	132.1649	367.8351
AMP	Ligation ins	NoAMP	No transfor	-80.5	29.68024	3.83569	0.32102	0.05	0	-198.335	37.33512
AMP	Ligation ins	NoAMP	Control	7	29.68024	0.33354	1	0.05	0	-110.835	124.8351
AMP	Ligation ins	NoAMP	Ligation ins	2.5	29.68024	0.11912	1	0.05	0	-115.335	120.3351
AMP	Ligation ins	NoAMP	CH3	21	29.68024	1.00061	0.99971	0.05	0	-96.8351	138.8351
AMP	Ligation ins	NoAMP	MTC5	26.5	29.68024	1.26268	0.99778	0.05	0	-91.3351	144.3351
AMP	Ligation ins	NoAMP	AdoHcy	11.5	29.68024	0.54796	1	0.05	0	-106.335	129.3351
AMP	Ligation ins	AMP	No transfor	254	29.68024	12.10267	7.34E-05	0.05	1	136.1649	371.8351
AMP	Ligation ins	AMP	Control	4	29.68024	0.19059	1	0.05	0	-113.835	121.8351
AMP	CH3	NoAMP	No transfor	-76	29.68024	3.62127	0.38807	0.05	0	-193.835	41.83512
AMP	CH3	NoAMP	Control	11.5	29.68024	0.54796	1	0.05	0	-106.335	129.3351
AMP	CH3	NoAMP	Ligation ins	7	29.68024	0.33354	1	0.05	0	-110.835	124.8351
AMP	CH3	NoAMP	CH3	25.5	29.68024	1.21503	0.9984	0.05	0	-92.3351	143.3351
AMP	CH3	NoAMP	MTC5	31	29.68024	1.4771	0.99221	0.05	0	-86.8351	148.8351
AMP	CH3	NoAMP	AdoHcy	16	29.68024	0.76237	0.99998	0.05	0	-101.835	133.8351
AMP	CH3	AMP	No transfor	258.5	29.68024	12.31709	6.12E-05	0.05	1	140.6649	376.3351
AMP	CH3	AMP	Control	8.5	29.68024	0.40501	1	0.05	0	-109.335	126.3351
AMP	CH3	AMP	Ligation ins	4.5	29.68024	0.21442	1	0.05	0	-113.335	122.3351
AMP	MTC5	NoAMP	No transfor	-100	29.68024	4.76483	0.12682	0.05	0	-217.835	17.83512
AMP	MTC5	NoAMP	Control	-12.5	29.68024	0.5956	1	0.05	0	-130.335	105.3351
AMP	MTC5	NoAMP	Ligation ins	-17	29.68024	0.81002	0.99996	0.05	0	-134.835	100.8351
AMP	MTC5	NoAMP	CH3	1.5	29.68024	0.07147	1	0.05	0	-116.335	119.3351
AMP	MTC5	NoAMP	MTC5	7	29.68024	0.33354	1	0.05	0	-110.835	124.8351
AMP	MTC5	NoAMP	AdoHcy	-8	29.68024	0.38119	1	0.05	0	-125.835	109.8351
AMP	MTC5	AMP	No transfor	234.5	29.68024	11.17353	1.64E-04	0.05	1	116.6649	352.3351
AMP	MTC5	AMP	Control	-15.5	29.68024	0.73855	0.99998	0.05	0	-133.335	102.3351
AMP	MTC5	AMP	Ligation ins	-19.5	29.68024	0.92914	0.99986	0.05	0	-137.335	98.33512
AMP	MTC5	AMP	CH3	-24	29.68024	1.14356	0.99905	0.05	0	-141.835	93.83512
AMP	AdoHcy	NoAMP	No transfor	-334.5	29.68024	15.93836	4.14E-06	0.05	1	-452.335	-216.665
AMP	AdoHcy	NoAMP	Control	-247	29.68024	11.76913	9.73E-05	0.05	1	-364.835	-129.165
AMP	AdoHcy	NoAMP	Ligation ins	-251.5	29.68024	11.98355	8.11E-05	0.05	1	-369.335	-133.665
AMP	AdoHcy	NoAMP	CH3	-233	29.68024	11.10206	1.75E-04	0.05	1	-350.835	-115.165
AMP	AdoHcy	NoAMP	MTC5	-227.5	29.68024	10.83999	2.21E-04	0.05	1	-345.335	-109.665
AMP	AdoHcy	NoAMP	AdoHcy	-242.5	29.68024	11.55472	1.17E-04	0.05	1	-360.335	-124.665
AMP	AdoHcy	AMP	No transfor	0	29.68024	0	1	0.05	0	-117.835	117.835
AMP	AdoHcy	AMP	Control	-250	29.68024	11.91208	8.61E-05	0.05	1	-367.835	-132.165
AMP	AdoHcy	AMP	Ligation ins	-254	29.68024	12.10267	7.34E-05	0.05	1	-371.835	-136.165
AMP	AdoHcy	AMP	CH3	-258.5	29.68024	12.31709	6.12E-05	0.05	1	-376.335	-140.665
AMP	AdoHcy	AMP	MTC5	-234.5	29.68024	11.17353	1.64E-04	0.05	1	-352.335	-116.665

Table S11. Two-way ANOVA results of BL21 with and without modified plasmids on control and kanamycin plates (statistically significant results highlighted in red).

Antibiotic	Modificatio	Antibiotic	Modificatio	MeanDiff	SEM	q Value	Prob	Alpha	Sig	LCL	UCL
NoKan	Control	NoKan	No transfor	-58	23.7364	3.45564	0.44536	0.05	0	-152.237	36.23716
NoKan	Ligation ins	NoKan	No transfor	-73	23.7364	4.34934	0.19544	0.05	0	-167.237	21.23716
NoKan	Ligation ins	NoKan	Control	-15	23.7364	0.8937	0.9999	0.05	0	-109.237	79.23716
NoKan	CH3	NoKan	No transfor	-96	23.7364	5.71968	0.04447	0.05	1	-190.237	-1.76284
NoKan	CH3	NoKan	Control	-38	23.7364	2.26404	0.87971	0.05	0	-132.237	56.23716
NoKan	CH3	NoKan	Ligation ins	-23	23.7364	1.37034	0.99567	0.05	0	-117.237	71.23716
NoKan	MTC5	NoKan	No transfor	-70.5	23.7364	4.20039	0.22686	0.05	0	-164.737	23.73716
NoKan	MTC5	NoKan	Control	-12.5	23.7364	0.74475	0.99998	0.05	0	-106.737	81.73716
NoKan	MTC5	NoKan	Ligation ins	2.5	23.7364	0.14895	1	0.05	0	-91.7372	96.73716
NoKan	MTC5	NoKan	CH3	25.5	23.7364	1.51929	0.99036	0.05	0	-68.7372	119.7372
NoKan	AdoHcy	NoKan	No transfor	-73	23.7364	4.34934	0.19544	0.05	0	-167.237	21.23716
NoKan	AdoHcy	NoKan	Control	-15	23.7364	0.8937	0.9999	0.05	0	-109.237	79.23716
NoKan	AdoHcy	NoKan	Ligation ins	0	23.7364	0	1	0.05	0	-94.2372	94.23716
NoKan	AdoHcy	NoKan	CH3	23	23.7364	1.37034	0.99567	0.05	0	-71.2372	117.2372
NoKan	AdoHcy	NoKan	MTC5	-2.5	23.7364	0.14895	1	0.05	0	-96.7372	91.73716
Kan	No transfor	NoKan	No transfor	-330.5	23.7364	19.69117	2.83E-07	0.05	1	-424.737	-236.263
Kan	No transfor	NoKan	Control	-272.5	23.7364	16.23554	3.42E-06	0.05	1	-366.737	-178.263
Kan	No transfor	NoKan	Ligation ins	-257.5	23.7364	15.34184	6.18E-06	0.05	1	-351.737	-163.263
Kan	No transfor	NoKan	CH3	-234.5	23.7364	13.9715	1.63E-05	0.05	1	-328.737	-140.263
Kan	No transfor	NoKan	MTC5	-260	23.7364	15.49079	5.59E-06	0.05	1	-354.237	-165.763
Kan	No transfor	NoKan	AdoHcy	-257.5	23.7364	15.34184	6.18E-06	0.05	1	-351.737	-163.263
Kan	Control	NoKan	No transfor	-79.5	23.7364	4.73661	0.13068	0.05	0	-173.737	14.73716
Kan	Control	NoKan	Control	-21.5	23.7364	1.28097	0.9975	0.05	0	-115.737	72.73716
Kan	Control	NoKan	Ligation ins	-6.5	23.7364	0.38727	1	0.05	0	-100.737	87.73716
Kan	Control	NoKan	CH3	16.5	23.7364	0.98307	0.99976	0.05	0	-77.7372	110.7372
Kan	Control	NoKan	MTC5	-9	23.7364	0.53622	1	0.05	0	-103.237	85.23716
Kan	Control	NoKan	AdoHcy	-6.5	23.7364	0.38727	1	0.05	0	-100.737	87.73716
Kan	Control	Kan	No transfor	251	23.7364	14.95457	8.09E-06	0.05	1	156.7628	345.2372
Kan	Ligation ins	NoKan	No transfor	-82	23.7364	4.88556	0.11144	0.05	0	-176.237	12.23716
Kan	Ligation ins	NoKan	Control	-24	23.7364	1.42992	0.99394	0.05	0	-118.237	70.23716
Kan	Ligation ins	NoKan	Ligation ins	-9	23.7364	0.53622	1	0.05	0	-103.237	85.23716
Kan	Ligation ins	NoKan	CH3	14	23.7364	0.83412	0.99995	0.05	0	-80.2372	108.2372
Kan	Ligation ins	NoKan	MTC5	-11.5	23.7364	0.68517	0.99999	0.05	0	-105.737	82.73716
Kan	Ligation ins	NoKan	AdoHcy	-9	23.7364	0.53622	1	0.05	0	-103.237	85.23716
Kan	Ligation ins	Kan	No transfor	248.5	23.7364	14.80562	8.99E-06	0.05	1	154.2628	342.7372
Kan	Ligation ins	Kan	Control	-2.5	23.7364	0.14895	1	0.05	0	-96.7372	91.73716
Kan	CH3	NoKan	No transfor	-102.5	23.7364	6.10695	0.02884	0.05	1	-196.737	-8.26284
Kan	CH3	NoKan	Control	-44.5	23.7364	2.65131	0.75478	0.05	0	-138.737	49.73716
Kan	CH3	NoKan	Ligation ins	-29.5	23.7364	1.75761	0.97289	0.05	0	-123.737	64.73716
Kan	CH3	NoKan	CH3	-6.5	23.7364	0.38727	1	0.05	0	-100.737	87.73716
Kan	CH3	NoKan	MTC5	-32	23.7364	1.90656	0.95424	0.05	0	-126.237	62.23716
Kan	CH3	NoKan	AdoHcy	-29.5	23.7364	1.75761	0.97289	0.05	0	-123.737	64.73716
Kan	CH3	Kan	No transfor	228	23.7364	13.58423	2.20E-05	0.05	1	133.7628	322.2372
Kan	CH3	Kan	Control	-23	23.7364	1.37034	0.99567	0.05	0	-117.237	71.23716
Kan	CH3	Kan	Ligation ins	-20.5	23.7364	1.22139	0.99833	0.05	0	-114.737	73.73716
Kan	MTC5	NoKan	No transfor	-113.5	23.7364	6.76232	0.01388	0.05	1	-207.737	-19.2628
Kan	MTC5	NoKan	Control	-55.5	23.7364	3.30669	0.50033	0.05	0	-149.737	38.73716
Kan	MTC5	NoKan	Ligation ins	-40.5	23.7364	2.41299	0.83631	0.05	0	-134.737	53.73716
Kan	MTC5	NoKan	CH3	-17.5	23.7364	1.04265	0.99958	0.05	0	-111.737	76.73716
Kan	MTC5	NoKan	MTC5	-43	23.7364	2.56194	0.78683	0.05	0	-137.237	51.23716
Kan	MTC5	NoKan	AdoHcy	-40.5	23.7364	2.41299	0.83631	0.05	0	-134.737	53.73716
Kan	MTC5	Kan	No transfor	217	23.7364	12.92885	3.70E-05	0.05	1	122.7628	311.2372
Kan	MTC5	Kan	Control	-34	23.7364	2.02572	0.93421	0.05	0	-128.237	60.23716
Kan	MTC5	Kan	Ligation ins	-31.5	23.7364	1.87677	0.95852	0.05	0	-125.737	62.73716
Kan	MTC5	Kan	CH3	-11	23.7364	0.65538	0.99999	0.05	0	-105.237	83.23716
Kan	AdoHcy	NoKan	No transfor	-330.5	23.7364	19.69117	2.83E-07	0.05	1	-424.737	-236.263
Kan	AdoHcy	NoKan	Control	-272.5	23.7364	16.23554	3.42E-06	0.05	1	-366.737	-178.263
Kan	AdoHcy	NoKan	Ligation ins	-257.5	23.7364	15.34184	6.18E-06	0.05	1	-351.737	-163.263
Kan	AdoHcy	NoKan	CH3	-234.5	23.7364	13.9715	1.63E-05	0.05	1	-328.737	-140.263
Kan	AdoHcy	NoKan	MTC5	-260	23.7364	15.49079	5.59E-06	0.05	1	-354.237	-165.763
Kan	AdoHcy	NoKan	AdoHcy	-257.5	23.7364	15.34184	6.18E-06	0.05	1	-351.737	-163.263
Kan	AdoHcy	Kan	No transfor	0	23.7364	0	1	0.05	0	-94.2372	94.23716
Kan	AdoHcy	Kan	Control	-251	23.7364	14.95457	8.09E-06	0.05	1	-345.237	-156.763
Kan	AdoHcy	Kan	Ligation ins	-248.5	23.7364	14.80562	8.99E-06	0.05	1	-342.737	-154.263
Kan	AdoHcy	Kan	CH3	-228	23.7364	13.58423	2.20E-05	0.05	1	-322.237	-133.763
Kan	AdoHcy	Kan	MTC5	-217	23.7364	12.92885	3.70E-05	0.05	1	-311.237	-122.763

Table S12. Two-way ANOVA results of BL21 and DH5α transformed with and without modified eGFP-AmpR containing plasmids on ampicillin plates (statistically significant results highlighted in red).

Bacterial subspecies	Plasmid modification	Bacterial subspecies	Plasmid modification	MeanDiff	SEM	q Value	Prob	Alpha	Sig	LCL	UCL
DH5a	No transformation	DH5a	Control	-269	17.96602	21.17461	2.55E-07	0.05	1	-329.346	-208.654
DH5a	AdoHcy-azide	DH5a	Control	-29.6667	17.96602	2.33524	0.58399	0.05	0	-90.0131	30.67975
DH5a	AdoHcy-azide	DH5a	No transformation	239.3333	17.96602	18.83937	1.88E-07	0.05	1	178.9869	299.6798
BL21	Control	DH5a	Control	-0.66667	17.96602	0.05248	1	0.05	0	-61.0131	59.67975
BL21	Control	DH5a	No transformation	268.3333	17.96602	21.12213	2.55E-07	0.05	1	207.9869	328.6798
BL21	Control	DH5a	AdoHcy-azide	29	17.96602	2.28276	0.6053	0.05	0	-31.3464	89.34642
BL21	No transformation	DH5a	Control	-269	17.96602	21.17461	2.55E-07	0.05	1	-329.346	-208.654
BL21	No transformation	DH5a	No transformation	0	17.96602	0	1	0.05	0	-60.3464	60.34642
BL21	No transformation	DH5a	AdoHcy-azide	-239.333	17.96602	18.83937	1.88E-07	0.05	1	-299.68	-178.987
BL21	No transformation	BL21	Control	-268.333	17.96602	21.12213	2.55E-07	0.05	1	-328.68	-207.987
BL21	AdoHcy-azide	DH5a	Control	-47.6667	17.96602	3.75213	0.15724	0.05	0	-108.013	12.67975
BL21	AdoHcy-azide	DH5a	No transformation	221.3333	17.96602	17.42248	4.33E-07	0.05	1	160.9869	281.6798
BL21	AdoHcy-azide	DH5a	AdoHcy-azide	-18	17.96602	1.41689	0.90863	0.05	0	-78.3464	42.34642
BL21	AdoHcy-azide	BL21	Control	-47	17.96602	3.69965	0.16649	0.05	0	-107.346	13.34642
BL21	AdoHcy-azide	BL21	No transformation	221.3333	17.96602	17.42248	4.33E-07	0.05	1	160.9869	281.6798
